# Supplementary material for: How energy determines spatial localisation and copy number of molecules in neurons
Source: Nat Commun. 2025 Feb 7;16:1424. doi: 10.1038/s41467-025-56640-0 (PMC11802781; doi:10.1038/s41467-025-56640-0)
Supplement: Supplementary file 1 — Supplementary Information [file 41467_2025_56640_MOESM1_ESM.pdf]

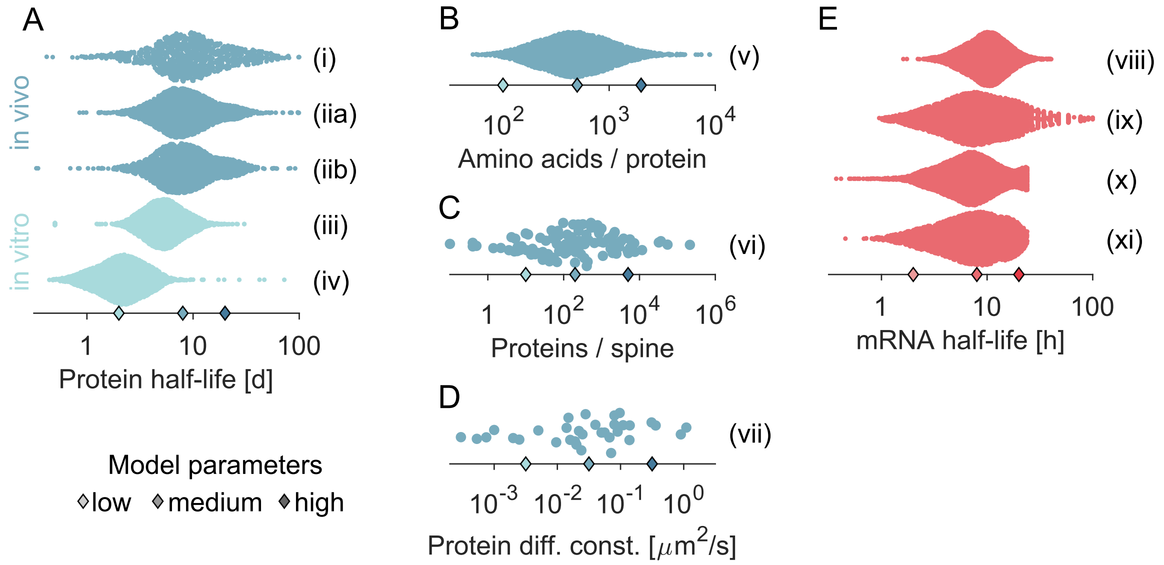

Supplementary Figure 1: Model parameters were sampled from available databases. **A** Protein lifetime (model parameters: 2, 8, 20d). Measurements performed in vitro (light blue) show lower protein lifetimes than in vivo (dark blue). Data sources are i[1], ii[2] (iia: cortex homogenate, iib: cerebellum homogenate), iii[3], iv[4]. **B** Protein size in amino acids (100, 500, 2000). Data source v is[2]. **C** Protein copy numbers per spine (10, 200, 5000). Data source vi is[5]. **D** Protein diffusion constants ($10^{-2.5}$, $10^{-1.5}$, $10^{-0.5}\mu m^2$/s). Data sources vii are reported in Supplementary Table 1. **E** mRNA half-life (2, 8, 20h). Data sources are viii[6], ix[7], x[8], xi[9].

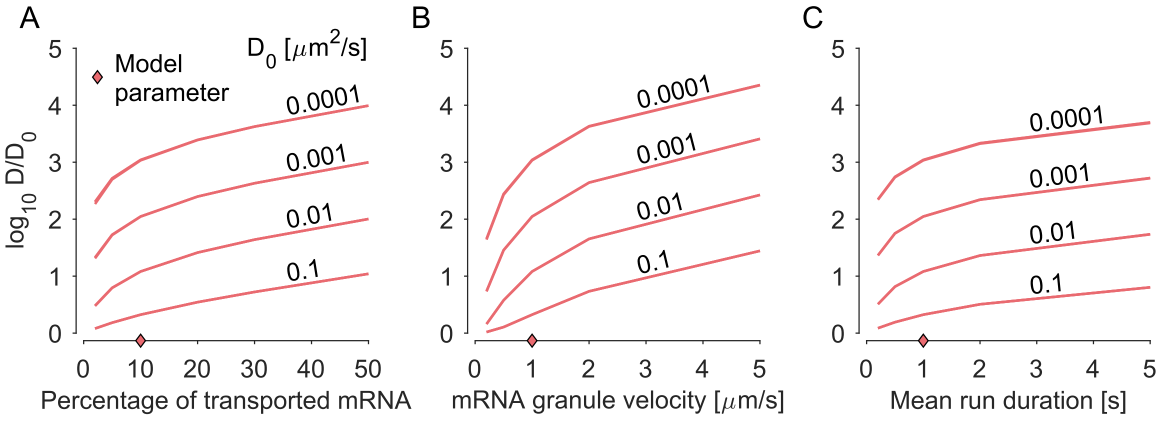

Supplementary Figure 2: Effect of transport model parameters on mRNA mobility. Ensemble mRNA diffusion constants were calculated for a range of transport model parameters, each of which was varied independently. We repeated this for a range of mRNA diffusion constants and half-lives. The mRNA half-life did not affect the fitted ensemble mRNA diffusion constant. In each panel, the ensemble diffusion constants are shown normalised with the passive mRNA diffusion constants. The varied transport parameters include the **A** percentage of transported mRNAs, **B** mRNA granule velocity, **C** average run duration (which equals the inverse switching rate β, see Supplementary Notes). The value of each parameter used throughout this work and in the other two panels is highlighted with a red diamond.

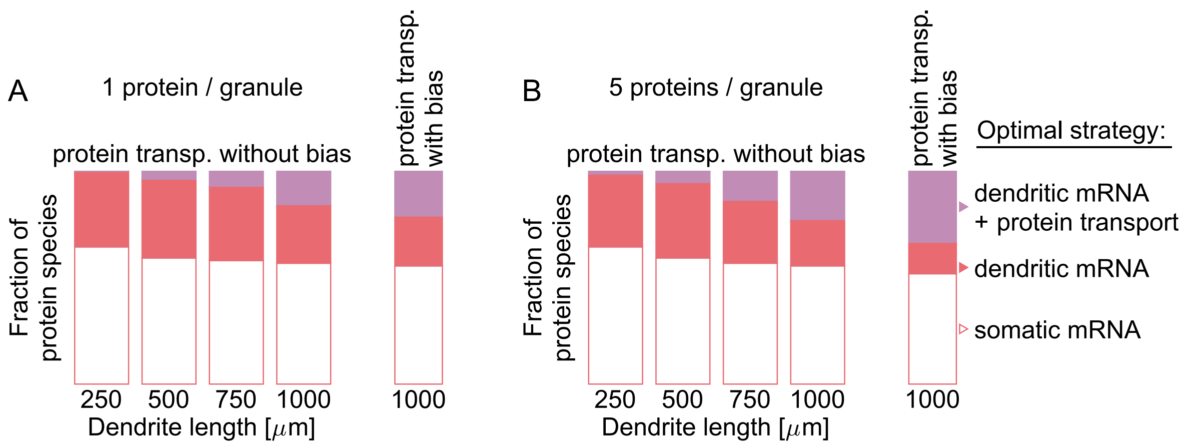

Supplementary Figure 3: Protein transport in small granules is energy efficient only as an 'add-on' to mRNA transport and solely in long dendrites. For each synthetic protein species within our parameter space we computed the total cost for four trafficking options: 1) somatic mRNA with no active transport at all (red, empty), 2) somatic mRNA with protein transport in absence of mRNA transport (blue, empty), 3) dendritic mRNA with mRNA transport but no protein transport (red, filled), and 4) dendritic mRNA with mRNA and protein transport (violet, filled). In dendrites of increasing length, we then computed which of the four possible transport schemes was energetically optimal for each synthetic protein species. In **A** we show the summary distribution per dendrite length. Somatic mRNA with protein transport but no mRNA transport was never optimal, therefore we omit the associated bar (blue, empty). To evaluate the effect of anterogradely biased protein transport, we added a net velocity of $\nu_p = 0.001 \mu m/s$ to our simulations and found that the preference for strategies remains largely unchanged (right bar). In **B**, we show the effect of transporting proteins in granules, reducing the transport cost proportional to the granule size. We find that a granule size of 90 proteins makes some species prefer protein transport over mRNA transport (empty blue bar appears from 500μm on). If a net protein velocity of $\nu_p = 0.001\mu m/s$ is applied, this effect becomes visible already for 60 proteins per granule.

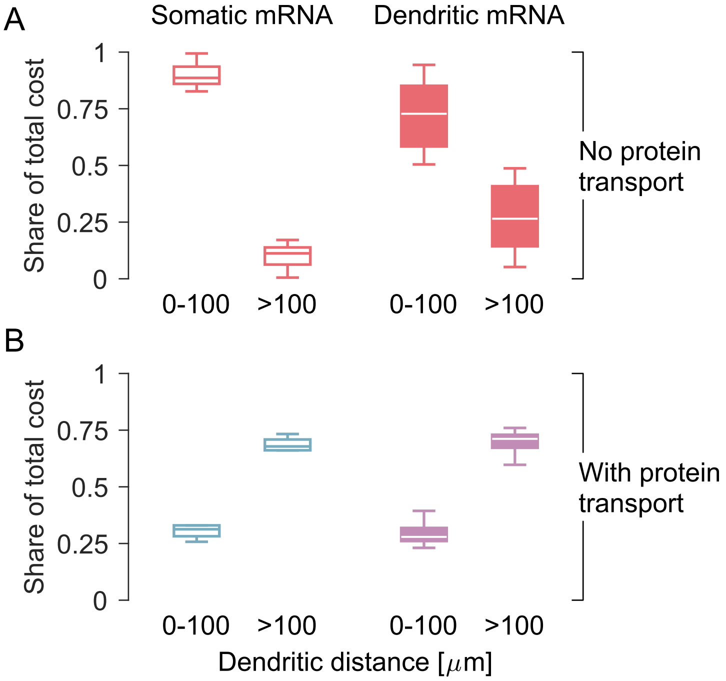

Supplementary Figure 4: Active protein transport shifts cost towards distal dendritic compartments (mRNA transport does not), making it incompatible with Figure 1F Total metabolic costs were calculated within and beyond the first 100μm for the entire parameter space of synthetic protein species, using a 1000μm long dendrite. Proteins either **A** moved purely diffusive or **B** were transported actively. In each row, all protein species rely on somatic (left) or dendritic (right) mRNA with mRNA transport. Each panel shows the share of total cost occurring before and after 100μm dendritic distance. All boxplots indicate median, quartiles and 1.5x interquartile ranges.

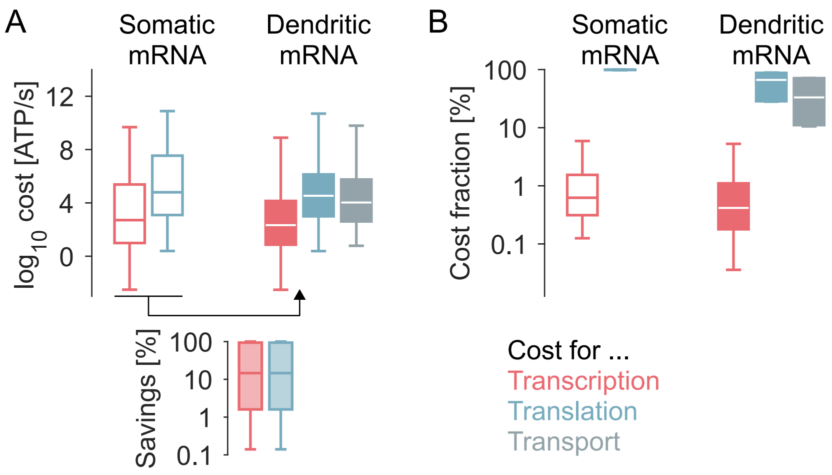

Supplementary Figure 5: Analysis of individual cost factors. A Predicted transcription (red), translation (blue), and transport cost (grey) if all protein species in our parameter space rely on somatic or dendritic mRNA. Bottom: translation and transcription cost savings per species if mRNAs are transported into dendrites versus staying in the soma. To determine if a protein species prefers dendritic mRNA localisation, these savings must be compared with the additional cost of mRNA transport. **B** Same as in A, but we normalised individual cost factors with the total costs for each species. The resulting cost fraction describes the contribution of each cost factor to the total costs. Results for A and B were calculated on a 1000μm long dendritic segment. All boxplots indicate median, quartiles and 1.5x interquartile ranges.

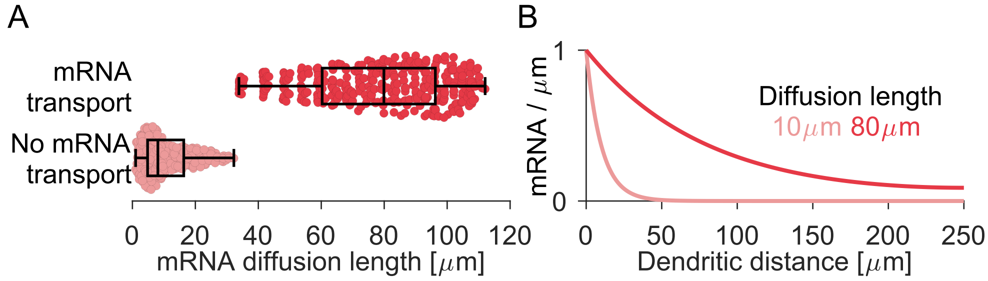

Supplementary Figure 6: mRNA transport is necessary to bring mRNAs away from soma and into dendrites. A Diffusion lengths were computed for the range of biologically plausible mRNA diffusion constants and half-lives we used throughout this work. mRNA diffusion constants were sampled from within 10^{-4} to $10^{-2}\mu m^2$/s, and mRNA half-lives from within 2-20 hours (for the parameter ranges, see the Supplementary Notes in section 'Sampling of synthetic protein species', Supplementary Figure 1, and Supplementary Table 6). Boxplots indicate the median, quartiles and 1.5x interquartile ranges. **B** Exemplary normalised mRNA distributions obtained from diffusion lengths representing mRNAs with and without transport. Representative diffusion lengths were chosen from A. Importantly, the predicted range of mRNA distributions, represented by their diffusion lengths, matched very well with reported data on the spatial density of mRNAs along dendrites[9–14] and observation that mRNAs travel longer distances only via active transport along the dendrite, in line with[15]. Finally, our classification of mRNA localisation as 'somatic' or 'dendritic' is valid because mRNAs not transported along the dendrite are spatially confined to the soma and the very proximal dendrite.

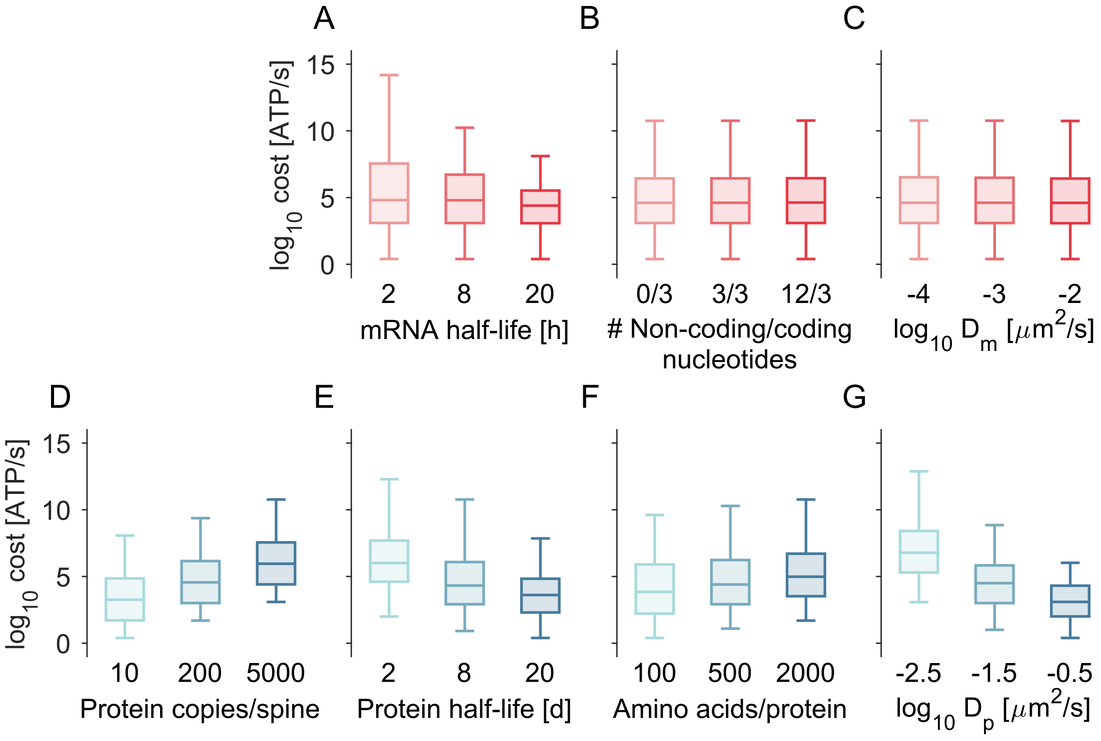

Supplementary Figure 7: Effects of mRNA and protein parameters on the predicted total energy budget if each synthetic protein species uses its most efficient localisation strategy. We computed the total costs for both localisation strategies (somatic and dendritic mRNA) and chose the cheaper alternative for every synthetic protein species in our parameter space. In each panel, we sorted the total costs per species along the parameters shown on the x-axis. Panels referring to mRNA parameters are shown in red, and protein parameters in blue. Simulations were performed on a 1000μm dendrite. In each panel, one parameter varies along the x-axis: **A** mRNA half-life, **B** mRNA length, **C** mRNA diffusion constant, **D** protein copy number per spine, **E** protein half-life, **F** protein length, **G** protein diffusion constant, All boxplots indicate median, quartiles and 1.5x interquartile ranges.

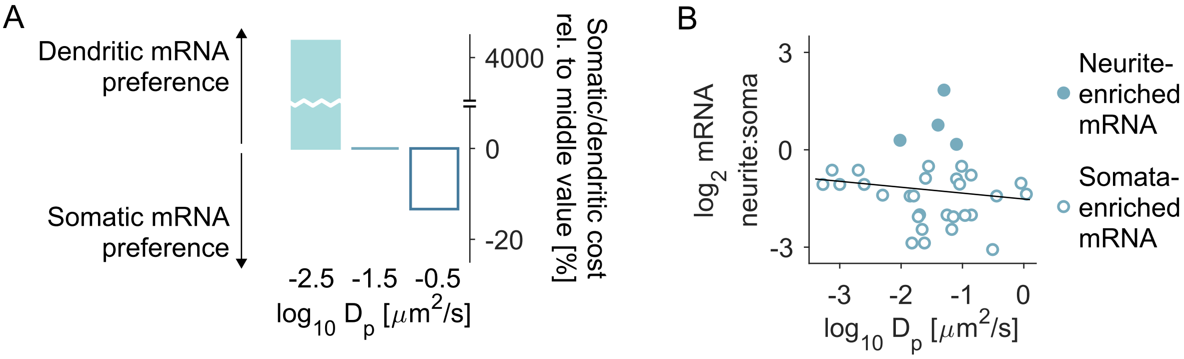

Supplementary Figure 8: Protein diffusion constants vs. mRNA localisation. A We performed the same analysis as in Figure 2 for the protein diffusion constant parameter in our model. We computed the total costs for all synthetic protein species in our parameter space (N=2187) with somatic and dendritic mRNA. Then, we divided the costs obtained with dendritic mRNA by those obtained with somatic mRNA for every synthetic protein species, giving us a measure of the relative efficiency of both strategies. Now, for each synthetic protein species, there are exactly two other proteins that differ only in protein diffusion constant because we sampled three values along each parameter dimension. Thereby, we created triplets of synthetic protein species corresponding to the protein diffusion constants $10^{-2.5}$, $10^{-1.5}$, $10^{-0.5}\mu m^2/s$. To examine how the relative efficiency of dendritic vs. somatic mRNA changes with the protein diffusion constant, we computed its change in every such triplet relative to the value corresponding to the medium protein diffusion constant $10^{-1.5}\mu m^2/s$. In **A**, we show the median change among all triplets. **B** Experimentally reported protein diffusion constants (N=32) from various sources were matched with mRNA enrichment scores from[16] (Supplementary Table 1). A linear least-squares fit with slope -0.200 (R^2=0.027) suggests a correlation between low diffusion constants and somatic mRNA enrichment, in line with the model prediction in **A**. As future studies increase the number of proteins with known diffusion constants, the significance of the linear fit could be further corroborated.

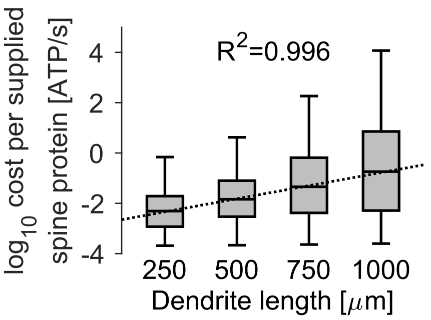

Supplementary Figure 9: Median total cost per supplied spine protein scale exponentially with dendrite length. Predicted total cost if every synthetic protein species uses the energetically most efficient mRNA localisation. Linear regression was performed on the distribution medians (R^2=0.997), and the result is shown as a dotted line. All boxplots indicate median, quartiles and 1.5x interquartile ranges.

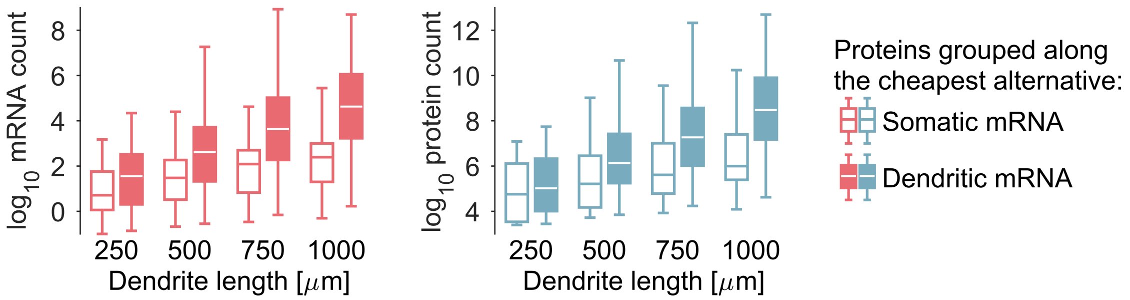

Supplementary Figure 10: Species preferring dendritic mRNA with mRNA transport are more abundant in mRNA and protein, independent of dendrite length. Predicted mRNA (left) and protein (right) abundances for synthetic protein species are grouped along the energetically preferred localisation pathway, either dendritic mRNA with mRNA transport (filled box) or somatic mRNA (empty box). Simulations were performed on dendrite lengths from 250 to 1000μm. All boxplots indicate median, quartiles and 1.5x interquartile ranges.

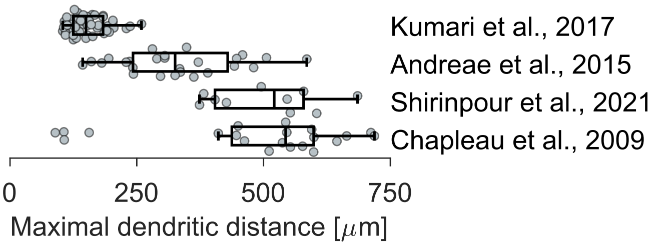

Supplementary Figure 11: Maximal path length for dendritic morphologies of cultured hippocampal rat neurons. To rule out drug effects, only neurons in the control condition were considered. We downloaded morphologies from neuromorpho.org and computed their maximal path lengths with the TREES toolbox[17]. Original resources are[18–21]

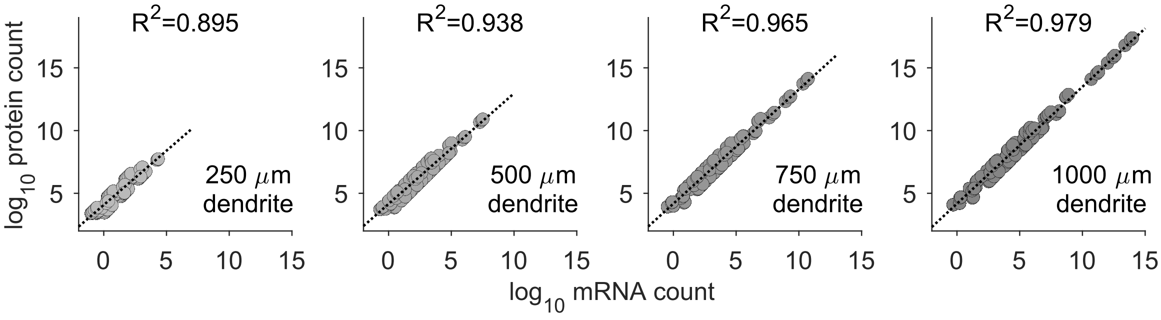

Supplementary Figure 12: Predicted total mRNA and protein numbers are highly correlated. Predicted total mRNA and protein counts are shown if each synthetic protein species relies on its energetically preferred localisation pathway, i.e., dendritic mRNA or somatic mRNA. Simulations were performed on dendrite lengths from 250 to 1000μm. Linear regressions (dotted lines) were applied to the abundance data, with the corresponding R^2-values printed at each panel's head.

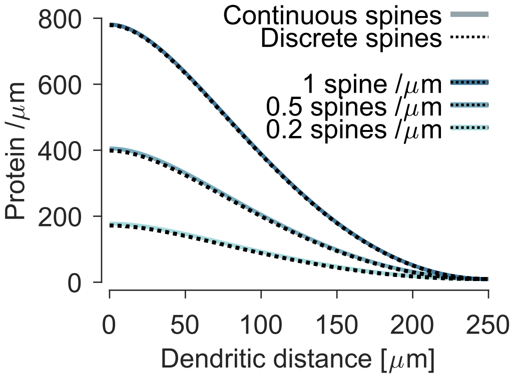

Supplementary Figure 13: Using discrete spines in the model does not change protein distributions if the spine locations are equidistant. For equidistant spine locations with various densities (0.2-1 spine per μm), the simulated protein distributions obtained with discrete (dashed lines) and continuous spine-dendrite interaction coincide. We simulated a synthetic protein species featuring dendritic mRNA on a 250μm long dendrite. Proteins had a half-life of 8d, a diffusion constant of 0.01μm^2/s, and a maximal spine capacity of 500, while the corresponding mRNAs had a half-life of 8h and a (passive) diffusion constant of 0.001μm^2/s.

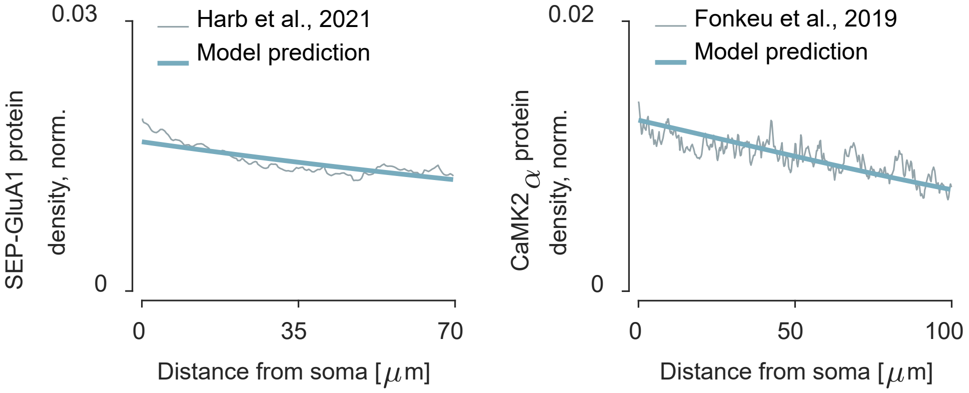

Supplementary Figure 14: Predicted dendritic distribution of *GluA1* protein (left), and *CamKII*α protein (right) compared to experimental data[11,22]. The dendritic distribution profile of *GluA1* (left) was captured from cultured mouse hippocampal neurons. The simulated dendritic protein profile was obtained on a 500μm long dendrite and normalised to the integrated density within the first 70μm to allow comparison with the data that are limited to this range. The predicted protein profile matches the experimentally reported density with distance from the soma[22]. Here, we used the following *GluA1* parameters: protein half-life 8.46 days[2], mRNA half-life 21.45 hours[9], amino acids per protein 907, non-coding nucleotides per transcript 2400[9], average copy number per spine 279.5[5] and diffusion constant 0.05$\mu m^2/s$[23]. The mRNA of *GluA1* is assumed to be primarily somatic, following the literature, e.g., ref.[9]. On the right, we show the experimentally obtained *CamKII* protein distribution[11] together with our corresponding model prediction from Figure 1C for comparison.

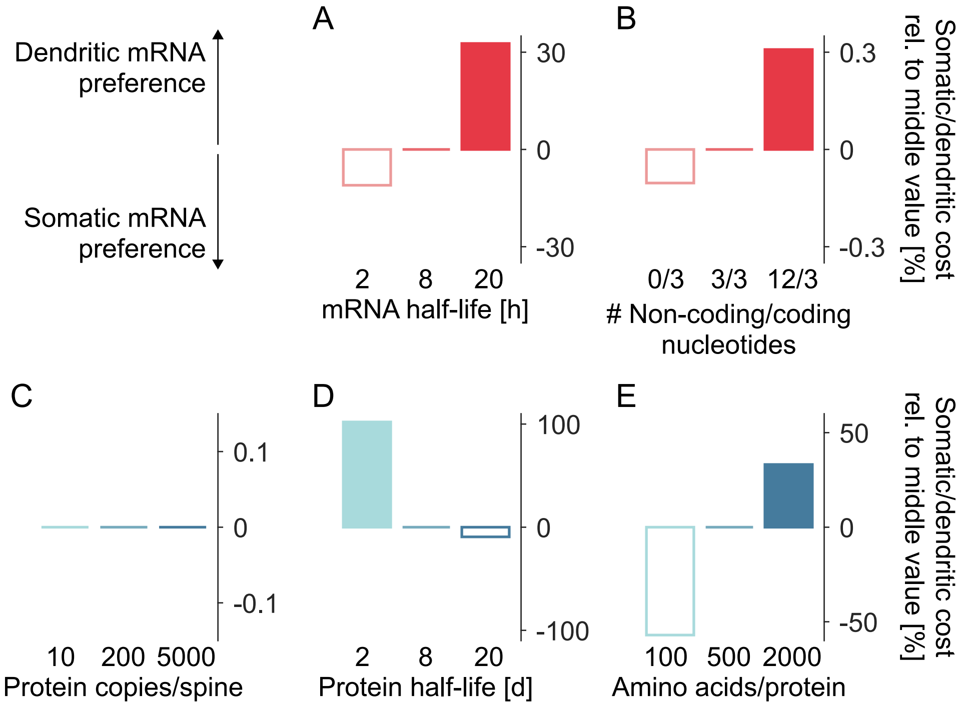

Supplementary Figure 15: Results in Figure 2 remain valid in the presence of a anterograde active transport bias (net forward mRNA velocity). We re-simulated the mRNA and protein distributions with their associated energetic costs assuming an anterograde net velocity of 0.0001μm/s for dendritic mRNAs. All other parameters were chosen as in Figure 2 in the main text. This shows that results shown in Figure 2 can be obtained equivalently in the presence of a net anterograde velocity in the active mRNA transport.

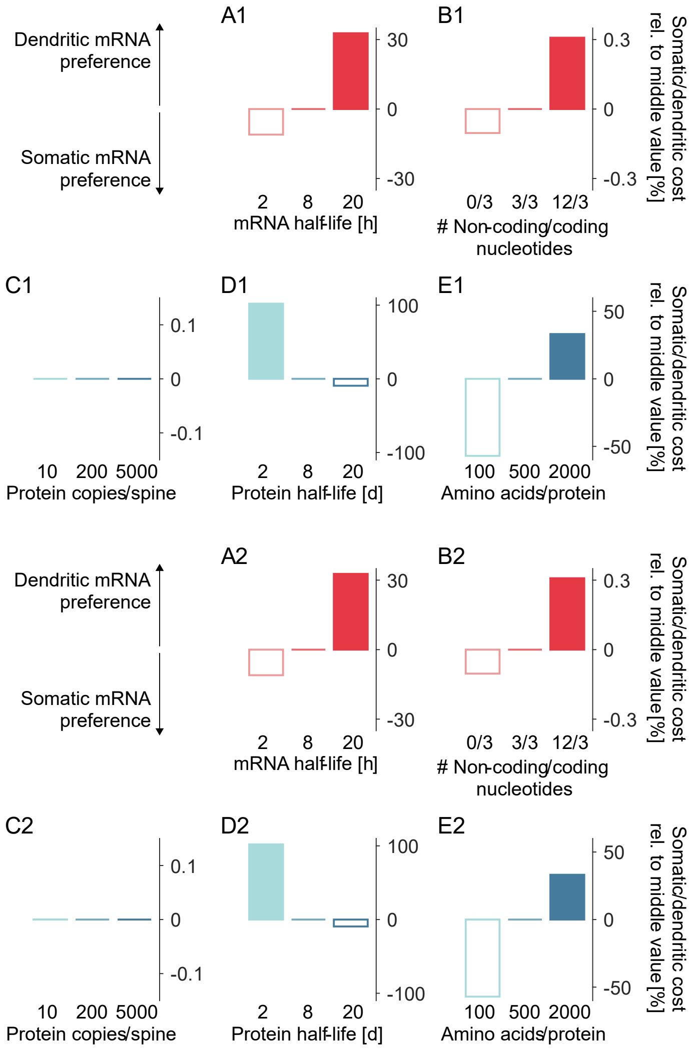

Supplementary Figure 16: Variations of the required spine supply rate ϕ do not affect our main results shown in Figure 2. We simulated the mRNA and protein distributions with their associated energetic costs assuming a spine supply rate ϕ of 0.85 (**A1-E1**) or 0.7 (**A2-E2**) instead of 0.95 which we used throughout the manuscript. Other than that, all parameters were chosen identically to Figure 2 in the main text. These results show that varying ϕ does not change the results in Figure 2 in our manuscript.

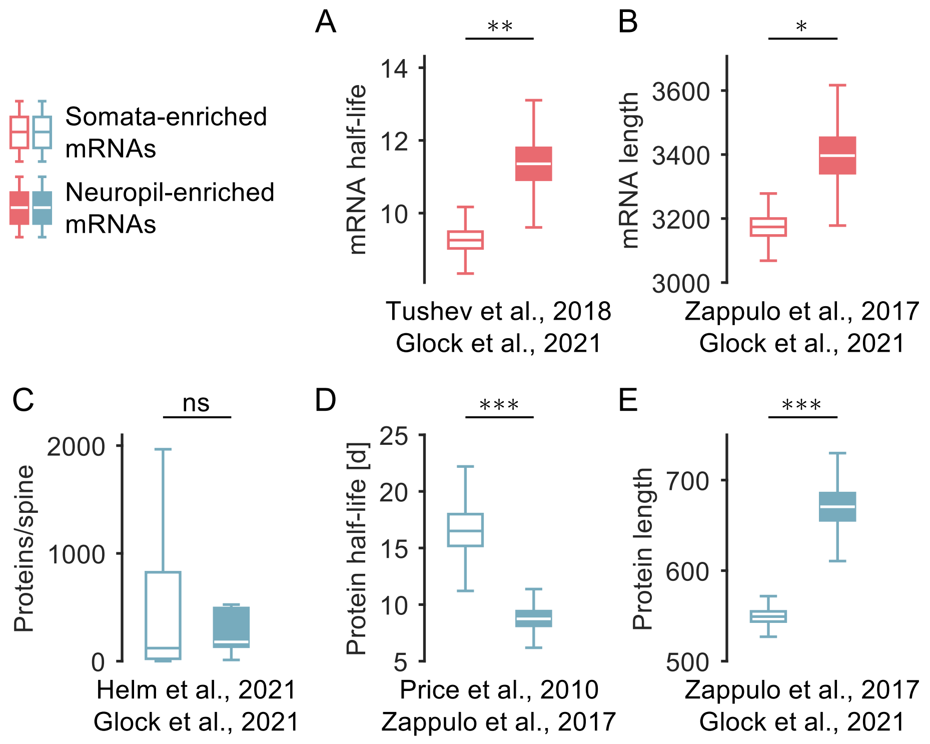

Supplementary Figure 17: Results presented in Figure 3 remain valid when considering different dataset pairs We confirmed the results presented in Figure 3 with different cross-matched datasets, these are data set pairs indicated in light gray in Supplementary Table 7 (dark gray data set pairs are shown in Figure 3). Analogously to Figure 3, we employ the following color code: mRNA (red) and protein (blue) parameters and their corresponding mRNA enrichment in neurites vs. somata were extracted from published datasets. In each panel the X axis label denotes the corresponding parameter. According to mRNA enrichment, we labeled database entries as neurite-enriched (filled boxes) or somata-enriched (empty boxes). **A** 3'-UTR isoform half-lives from[9] and corresponding enrichment scores from[24] (N_{somata}=319, $N_{neurite}$=94, p=3.6×10^{-3}, bootstrapped to 10k over mean); **B** Transcript length in nucleotide by[16] and mRNA enrichment of[24] (N_{somata}=2597, $N_{neurite}$=704, p=2.57×10^{-2}, bootstrapped to 10k over mean); **C** Protein counts per spine from our data[5] and mRNA enrichment from[24] (N_{somata}=43, $N_{neurite}$=15, $p = 2.82 \times 10^{-1}$); **D** Protein half-lives from[1] matched with mRNA enrichment scores of[16] (N_{somata}=194, $N_{neurite}$=61, p=1.24×10^{-4}, bootstrapped to 10k over mean); **E** Protein length in amino acids and mRNA enrichment scores from[16] matched with mRNA enrichement scores by[24] (N_{somata}=2597, $N_{neurite}$=704, p=4.32×10^{-6}, bootstrapped to 10k over mean). Boxplots indicate median, quartiles and 1.5x interquartile ranges. *($p < 0.05$), **($p < 0.01$), ***($p < 0.001$), two-sided Wilcoxon ranksum test, pairwise comparison within each panel.

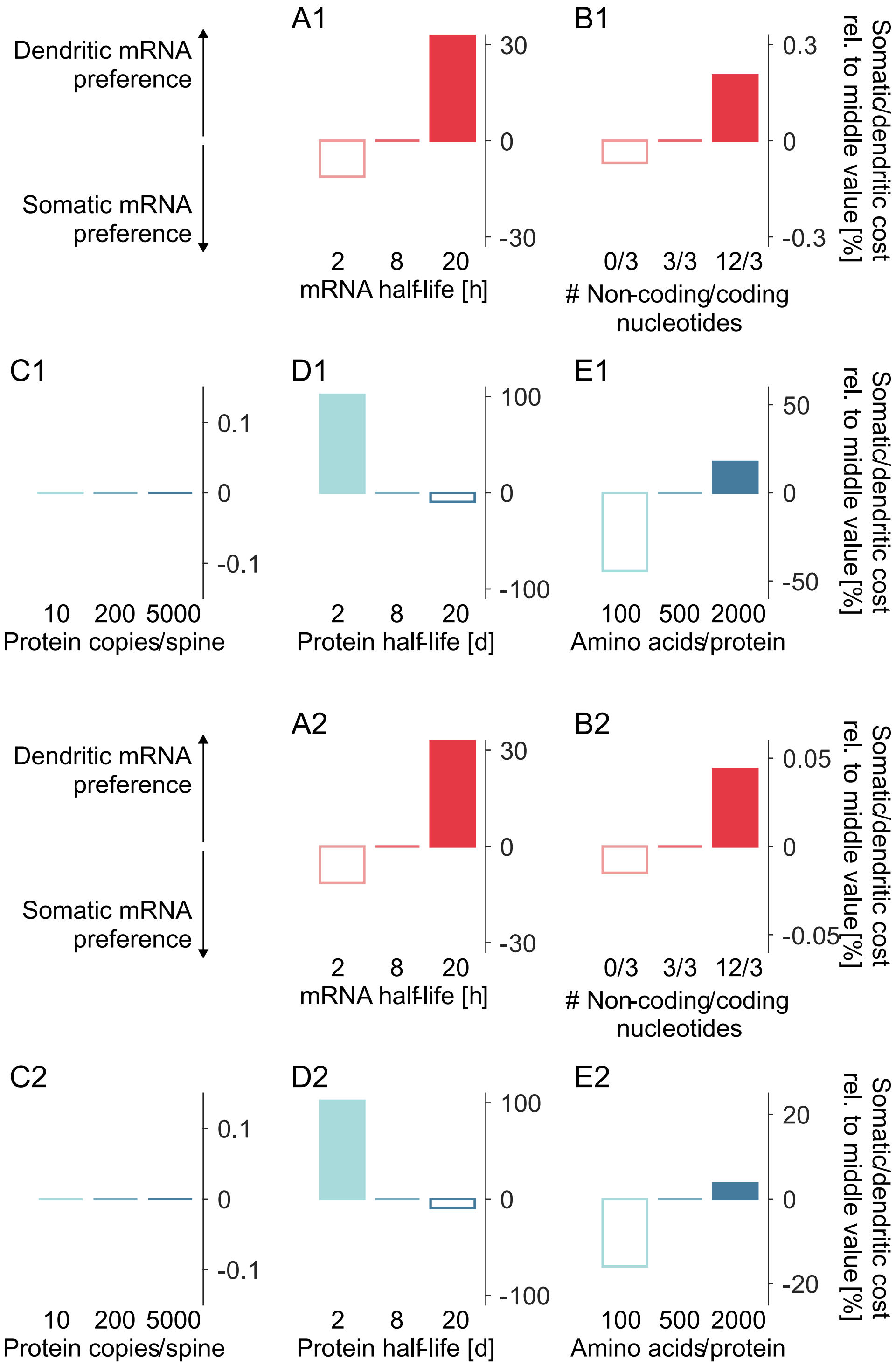

Supplementary Figure 18: Results in Figure 2 remain valid in the presence of multiple mRNAs per granule. We re-simulated the mRNA and protein distributions with their associated energetic costs assuming either 2 (A1-E1) or 10 (A2-E2) mRNAs per granule. All other parameters were chosen as in Figure 2 in the main text. This illustrates that the results shown in Figure 2 can be obtained equivalently in the presence of larger transport-competent mRNA granules.

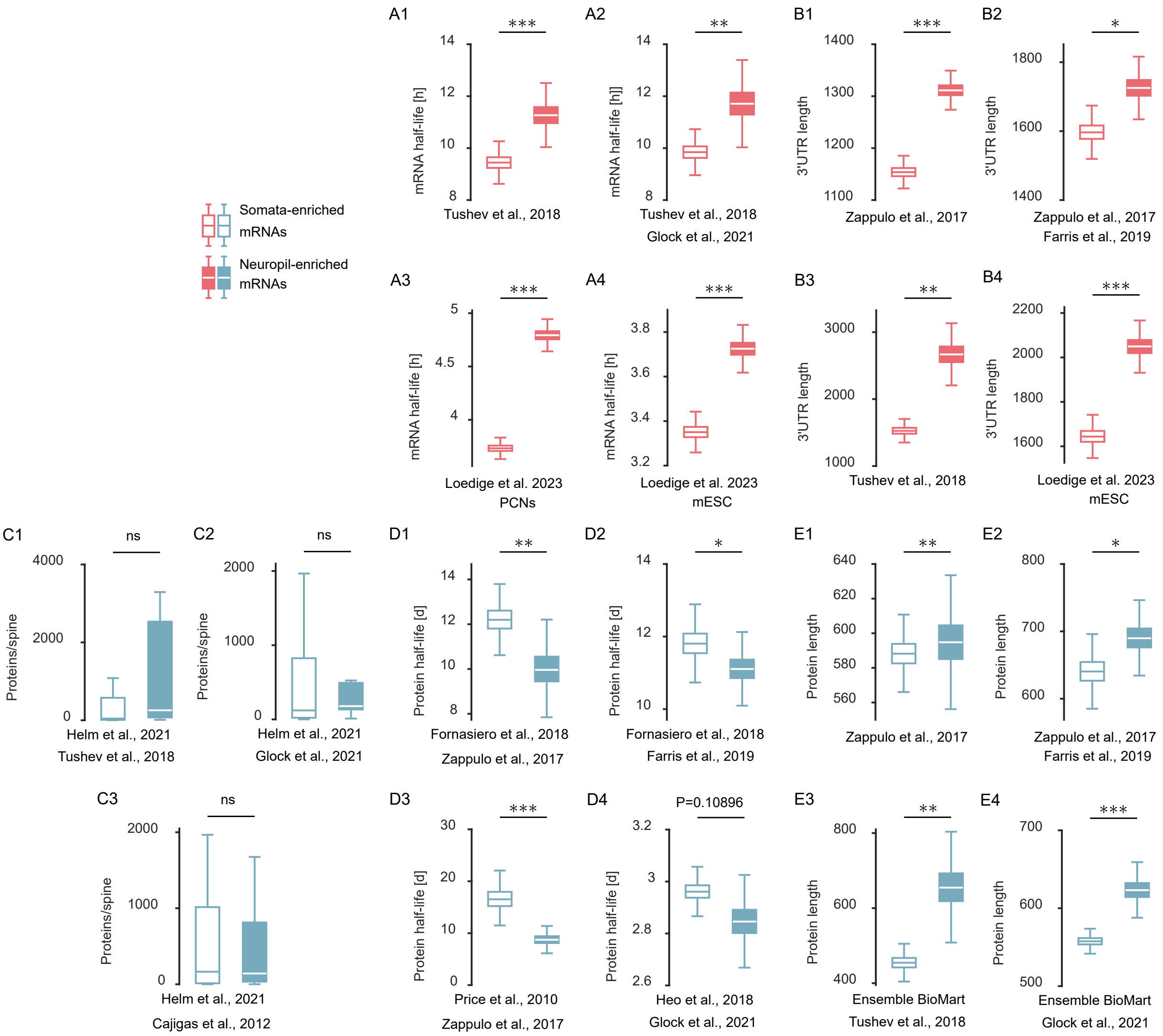

Supplementary Figure 19: Extended analysis of Figure 3 focusing on multiple database pairings for the same species Results presented in Figure 3 remain valid with various dataset pairs. By using the same species from an individual dataset or matching it with the dataset of the same species from another study in necessary cases (Supplementary Table 8), we show the results presented in Figure 3 and Supplementary Figure 17 remain the same. Analogously to Figure 3, we employ the following color code: mRNA (red) and protein (blue) parameters, and their corresponding mRNA enrichment in neurites vs. somata were extracted from published datasets. In each panel, the Y-axis label denotes the corresponding parameter. According to mRNA enrichment, we labeled database entries as neurite-enriched (filled boxes) or somata-enriched (empty boxes). **A1-A4** 3'-UTR isoform half-lives and corresponding enrichment scores[9,24,25] (N_{somata}=341, 319, 2086, 1962; $N_{neurite}$=201, 94, 2086, 1962; p=9.97×10^{-4}, 3.03×10^{-3}, 6.59×10^{-48}, 8.59×10^{-11}; bootstrapped to 10k over mean); **B1-B4** 3'-UTR length in nucleotide and mRNA enrichments[9,16,25,26] (N_{somata}=12894, 2427, 639, 1898; $N_{neurite}$=8408, 2363, 498, 1910; p=1.60×10^{-47}, 2.92×10^{-2}, 1.50×10^{-3}, 5.49×10^{-5}; bootstrapped to 10k over mean); **C1-C3** Protein counts per spine from our data[5] and mRNA enrichments from[9,24,27] (N_{somata}=8, 43, 17; $N_{neurite}$=3, 15, 17; p=4.97×10^{-1}, 2.83×10^{-1}, 9.04×10^{-1}); **D1-D4** Protein half-lives matched with corresponding mRNA enrichment scores[1,2,16,24,26,28] (N_{somata}=418, 486, 194, 1222; $N_{neurite}$=151, 546, 61, 281; p=1.90×10^{-3}, 4.61×10^{-2}, 1.25×10^{-4}, 1.08×10^{-1}; bootstrapped to 10k over mean); **E1-E4** Protein length in amino acids matched with corresponding mRNA enrichment scores[9,16,24,26] and Ensemble BioMart tool[29] (N_{somata}=5623, 933, 226, 5093; $N_{neurite}$=1406, 937, 116, 1782; p=4.98×10^{-3}, 3.61×10^{-2}, 6.78×10^{-3}, 7.96×10^{-4}; bootstrapped to 10k over mean). Boxplots indicate median, quartiles and 1.5x interquartile ranges. *($p < 0.05$), **($p < 0.01$), ***($p < 0.001$), two-sided Wilcoxon ranksum test, pairwise comparison within each panel.

Protein name	Gene name	log_2 mRNA neurite/soma (Zappulo et al., 2017)	D [μm^2/s]	Citation	Tissue
Acetylcholinesterase	*Ache*	-0.897	0.08	[30]	Xenopus myotomal muscle cells cultures
Actin	*Actb*	0.7611	0.04	[31]	Drosophila tracheal tubules
Clathrin light chain A	*Clta*	0.2924	0.0096	[32]	Synaptic terminal of retinal bipolar cells
D(1A) dopamine receptor	*Drd1a*	-1.0592	0.09	[23]	Rats hippocampal neuronal cultures
Ephrin type-B receptor 2	*Ephb2*	0.1685	0.08	[23]	Rats hippocampal neuronal cultures
GABA(A) receptor subunit alpha-1	*Gabra1*	-2.4533	0.022<br>0.067	[33]<br>[34]	Rats hippocampal neuronal cultures<br>Mice hippocampal neuronal cultures
GABA(A) receptor subunit alpha-2	*Gabra2*	-1.4235	0.36<br>0.016<br>0.014	[23]<br>[33]<br>[34]	Rats hippocampal neuronal cultures<br>Rats hippocampal neuronal cultures<br>Mice hippocampal neuronal cultures
GABA(A) receptor subunit alpha-5	*Gabra5*	-0.5078	0.097<br>0.028	[34]<br>[35]	Mice hippocampal neuronal cultures<br>Rats hippocampal neuronal cultures
GABA(A) receptor subunit gamma-2	*Gabrg2*	-2.8725	0.024<br>0.015	[36]<br>[35]	Rats hippocampal neuronal cultures<br>Rats hippocampal neuronal cultures
Glutamate receptor ionotropic, AMPA type subunit 1	*Gria1*	1.8386	0.05	[23]	Rats hippocampal neuronal cultures
Glutamate receptor ionotropic, AMPA type subunit 2	*Gria2*	-2.0088	0.14<br>0.056	[37]<br>[35]	Rats hippocampal neuronal cultures<br>Rats hippocampal neuronal cultures
Glutamate receptor ionotropic, NMDA 2A	*Grin2a*	-0.6279	0.002<br>0.0002979<br>0.00075	[23]<br>[38]<br>[39]	Rats hippocampal neuronal cultures<br>acute hippocampal slices obtained from adult Wistar rats<br>Rats hippocampal neuronal cultures
Glutamate receptor ionotropic, NMDA 2B	*Grin2b*	-1.0681	0.0025<br>0.0005322<br>0.001	[39]<br>[38]<br>[23]	Rats hippocampal neuronal cultures<br>acute hippocampal slices obtained from adult Wistar rats<br>Rat hippocampal neuronal cultures
Metabotropic glutamate receptor 5	*Grm5*	-0.8783	0.025	[40]	Rats hippocampal neuronal cultures
Voltage-gated potassium channel subunit Kv1.3	*Kcna3*	-3.074	0.31	[23]	Rats hippocampal neuronal cultures
Neural cell adhesion molecule L1	*L1cam*	-2.0215	0.11	[41]	surface of ND-7 neuroblastoma hybrid cells
Neurofascin	*Nfasc*	-2.0024	0.02	[42]	Rats hippocampal neuronal cultures
alpha-Neurexin 1	*Nrxn1a*	-2.0633	0.071	[43]	Rats and mice hippocampal neuronal cultures
beta-Neurexin 1	*Nrxn1b*	-2.0633	0.019	[43]	Rats and mice hippocampal neuronal cultures
Shank3	*Shank3*	-1.0284	0.9	[44]	Rats hippocampal neuronal cultures
Synapsin 1	*Syn1*	-1.3661	1.1	[44]	Rats hippocampal neuronal cultures
Synaptophysin	*Syp*	-1.3975	0.005	[32]	synaptic terminal of retinal bipolar cells

Supplementary Table 1: Summary of curated passive protein diffusion constants matched with their corresponding mRNA enrichment scores from[16] after acquiring gene names from UniProt[45]. We used this table to analyse the effect of protein diffusion constant in localisation patterns considering optimal energy expenditure (Supplementary Figure 8B).

	Quantity of interest	Sample size		Somata-enriched	Neurite-enriched
		All	Matched		
**Zappulo et al., 2017** [16]<br>(Induced mouse neurons)	mRNA length<br>Protein Length<br>mRNA abundance<br>Protein abundance	34258	-	5623<br>5623<br>5623<br>852	1406<br>1406<br>1406<br>622
**Tushev et al., 2018** [9]<br>(Rats hippocampal neuronal cultures)	mRNA half-life	24435	-	341	201
**Fornasiero et al., 2018** [2]<br>(Mice brain cortex synaptosomes)<br>**(Zappulo et al., 2017)** [16]<br>(Induced mouse neurons)	Protein half-life	3731<br>(34258)	3659	418	151
**Helm et al., 2021** [5]<br>(Rats hippocampal neuronal cultures)<br>**(Zappulo et al., 2017)** [16]<br>(Induced mouse neurons)	Proteins per spine	109<br>(34258)	83	58	25
**Helm et al., 2021** [5]<br>(Rats hippocampal neuronal cultures)	Proteins per neuron	6204	-	-	-
**Perez et al., 2021** [46]<br>(Rats and mice hippocampal neuronal cultures)	mRNAs per neuron	14248	-	-	-
**Zeisel et al., 2015** [47]<br>(Mice somatosensory cortex or hippocampal)	mRNAs per neuron	19184	-	-	-

Supplementary Table 2: Summary of the databases used for Figures 3, 4. Columns highlight the quantity derived from each source, the maximal database size for that quantity, the number of gene matches among sources (if two datasets were to be combined), and the counts of mRNAs labelled somata-enriched and neurite-enriched.

p	$\mu\mathrm{m}^{-1}$	concentration of dendritic proteins	m	s^{-1}	concentration of dendritic mRNAs
D_p	$\mu\mathrm{m}^2$/s	protein diffusion constant	$\tilde{D}_m$	$\mu\mathrm{m}^2$/s	ensemble mRNA diffusion constant
λ_p	s^{-1}	protein degradation rate	λ_m	s^{-1}	mRNA degradation rate
p_{spine}	$\mu\mathrm{m}^{-1}$	concentration of protein in spines	τ	s^{-1}	protein synthesis rate per mRNA
u_p	s^{-1}	rate of protein integration in spine	ρ	$\mu\mathrm{m}^{-1}$	dendritic spine density
ν_p	s^{-1}	rate of proteins leaving spines	η_p	$\emptyset$	max. protein count per spine

Supplementary Table 3: Parameters used in (eq. 11) with units and descriptions.

		Initiation rate	
		2min^{-1}	5min^{-1}
Ratio	10%	0.003/s	0.008/s
	25%	0.008/s	0.021/s
	70%	0.023/s	0.058/s

Supplementary Table 4: Translation rates in protein per mRNA and second, calculated from the ratio of currently translated mRNA ('Ratio') and the initiation rate per translated mRNA and minute.

**mRNA transport model parameter**	**Value**	**References**
Fraction of transported mRNA granules θ	10%	refs.[13,48–56]
mRNA granule velocity v during the run phase	1μm/s	refs.[13,15,48,49,51,53,54,56–63]
Transport state exit rate β	1s	refs.[13,58,63–68]

Supplementary Table 5: Summary of the parameters used for the mRNA transport model. The references are discussed in context in the corresponding Supplementary Notes sections of identical names. We use the same values for the protein transport parameters, for which we argue in the section 'Capturing active protein transport in our model' based on work by[43,69–73].

Parameters	Sampled in Figure 2<br>Supplementary Figure 1	Citation
**mRNA half-life**<br>**[hours]**	2, 8, 20	[6–9]
**mRNA length**<br>**[#Non-coding/coding nucleotides]**	(3, 6, 15) * aa<br>[0/3, 3/3, 12/3]	[74–76]
**Protein copy number**	10, 200, 5000	[5]
**Protein half-life**<br>**[days]**	2, 8, 20	[1–4]
**Protein length**<br>**[amino acids]**	100, 500, 2000	[2]
**mRNA diffusion**<br>$[\mu m^2/s]$	10^{-4}, 10^{-3}, 10^{-2}	[10, 13, 15, 49, 51, 52, 55, 64, 77–82]
**Protein diffusion**<br>$[\mu m^2/s]$	$10^{-2.5}$, $10^{-1.5}$, $10^{-0.5}$	Supplementary Table S1

Supplementary Table 6: Sampled parameter space range considered for our main predictions in Figures 1, 2, 4. See Supplementary Figure 1 for the actual parameter distributions.

Parameter combination	Main data	Matched data	Eligible matches	
			Somata-enriched	Neurite-enriched
mRNA half-life vs Enrichment scores	Tushev et al. 2018[9]	Tushev et al. 2018[9]	341	201
		Glock et al. 2021[24]	319	94
		Zappulo et al. 2017[16]	210	22
mRNA length vs Enrichment scores	Zappulo et al. 2017[16]	Zappulo et al. 2017[16]	5623	1406
		Glock et al. 2021[24]	2597	704
		Tushev et al. 2018[9]	314	191
Protein copy number vs Enrichment scores	Helm et al. 2021[5]	Zappulo et al. 2017[16]	58	25
		Glock et al. 2021[24]	43	15
		Tushev et al. 2018[9]	8	3
Protein half-life vs Enrichment scores	Fornasiero et al. 2018[2]	Zappulo et al. 2017[16]	418	151
		Tushev et al. 2018[9]	313	200
		Glock et al. 2021[24]	221	27
	Price et al. 2010[1]	Zappulo et al. 2017[16]	194	61
		Glock et al. 2021[24]	95	14
		Tushev et al. 2018[9]	31	17
Protein length vs Enrichment scores	Zappulo et al. 2017[16]	Zappulo et al. 2017[16]	5623	1406
		Glock et al. 2021[24]	2597	704
		Tushev et al. 2018[9]	314	191

Supplementary Table 7: Dataset cross-match overview. Detailed overview of the databases used to verify our model predictions. For each parameter combination, we have cross-matched two databases, one with a reported parameter of interest and the other with mRNA localization score and categorized them into somata- or neurite-enriched groups. In Figure 3 we use database pairs highlighted in dark gray which were selected based on the higher number of eligible matches. Similarly in light gray are the alternative cross-matched pairs used for the cross-check in Supplementary Figure 17. We have chosen the database matches for illustration in Figure 3 and Supplementary Figure 17 which have a higher matching number, relatively balanced distribution among the somata- and neurite-enriched categories and considered additional author-provided filters (e.g. removing data flagged as non-significant or missing). For protein half-life, we chose to use data by[1] in Supplementary Figure 17 as an alternative validation.

Panels	Data	Cell type
A1	Tushev et al. 2018[9]	Rat hippocampal slices
A2	Tushev et al. 2018[9]<br>Glock et al. 2021[24]	Rat hippocampal slices<br>Rat hippocampal slices
A3	Loedige et al. 2023[25]	Mouse primary cortical neurons
A4	Loedige et al. 2023[25]	Mouse embryonic stem cells
B1	Zappulo et al. 2017[16]	Mouse mESC-derived neurons
B2	Zappulo et al. 2017[16]<br>Farris et al. 2019[26]	Mouse mESC-derived neurons<br>Mouse hippocampal slices
B3	Tushev et al. 2018[9]	Rat hippocampal slices
B4	Loedige et al. 2023[25]	Mouse embryonic stem cells
C1	Helm et al. 2021[5]<br>Tushev et al. 2018[9]	Rat cultured hippocampal neurons<br>Rat hippocampal slices
C2	Helm et al. 2021[5]<br>Glock et al. 2021[24]	Rat cultured hippocampal neurons<br>Rat hippocampal slices
C3	Helm et al. 2021[5]<br>Cajigas et al. 2012[27]	Rat cultured hippocampal neurons<br>Rat hippocampal slices
D1	Fornasiero et al. 2018[2]<br>Zappulo et al. 2017[16]	Mouse cortex synaptosomes<br>Mouse mESC-derived neurons
D2	Fornasiero et al. 2018[2]<br>Farris et al. 2019[26]	Mouse cortex synaptosomes<br>Mouse hippocampal slices
D3	Price et al. 2010[1]<br>Zappulo et al. 2017[16]	Mouse brain<br>Mouse mESC-derived neurons
D4	Heo et al. 2018[28]<br>Glock et al. 2021[24]	Rat cultured neurons<br>Rat hippocampal slices
E1	Zappulo et al. 2017[16]	Mouse mESC-derived neurons
E2	Zappulo et al. 2017[16]<br>Farris et al. 2019[26]	Mouse mESC-derived neurons<br>Mouse hippocampal slices
E3	Ensemble BioMart[29]<br>Tushev et al. 2018[9]	Rat genome assembly Rnor_6.0 (release 104)<br>Rat hippocampal slices
E4	Ensemble BioMart[29]<br>Glock et al. 2021[24]	Rat genome assembly Rnor_6.0 (release 104)<br>Rat hippocampal slices

Supplementary Table 8: References used in Supplementary Figure 19 and the cell type of the used datasets. For each subpanel, we only cross-matched the datasets within the same species.

Supplementary Notes

Capturing active mRNA transport in our model

Motor-mediated transport along microtubules in dendrites has been reported across numerous mRNA species. mRNAs are generally transported in specific granules consisting of one or more mRNA molecules accompanied by binding proteins[83]. These transport-competent granules can bind to kinesin and dynein motors, which move along microtubules and carry the attached cargo, which can be, f.e, organelles, mRNA transport granules or protein vesicles. Here, we focus on mRNA granules and protein vesicles. While the spatial arrangement of microtubules in dendrites is intricate[84], dendritic transport is broadly classified as either anterograde (away from the soma) or retrograde (toward the soma). Several mathematical models were suggested to formalise the 'tug-of-war'-like intracellular motor-mediated transport (e.g., refs.[11,85–88]). Here, we chose a model by[85] with three mobility states for mRNA granules in an idealised one-dimensional dendrite: anterograde transport, retrograde transport, and unbound diffusive. Following experimental observations[13,48], mRNA granules switch between different mobility states. Therefore, single granule trajectories contain periods of directed motion and diffusive or immobile phases. It was crucial to be able to assign energy costs to our transport model. Therefore, we split mRNA granules being currently transported from the non-transported rest. We refer to the non-transported mRNA granules as diffusive or resting, and this state covers, for example, mRNAs diffusing in the cytosol and those bound to organelles. Active mRNA transport has been extensively studied, so we were able to derive robust numbers for the instantaneous granule velocity, the duration of single runs, and the overall time spent in transport. With these values and estimates of the diffusivity of not-transported mRNAs, we can formulate our model (eq. 1 in the 'Results' section), which provides a good trade-off between biological accuracy and parameter robustness. Besides that, our model for dendritic mRNA dynamics has a further practical advantage. Under the assumption of symmetric transport mechanisms, i.e., similar amounts of mRNAs move anterogradely and retrogradely with identical speed and run duration, our model represents a one-dimensional random walk and can be represented by a single diffusion constant. The latter is called ensemble or effective diffusion constant. The fitted ensemble diffusion constants allow us to seamlessly integrate mRNA transport in our general framework to distribute mRNAs and proteins in dendrites and spines, implying that we can then directly quantify the effect of mRNA transport on the distribution of dendritic mRNA.

Symmetry of mRNA transport. In our model, mRNA transport is assumed to be overall symmetric, i.e., all parameters are the same for anterograde and retrograde states. mRNA granule movements are mediated by molecular motors moving towards the microtubules' plus-end (kinesins) and minus-end (dyneins). Many questions concerning the motor protein action, its efficacy and symmetry, the number and directionality of microtubular tracks (plus/minus ends), and their spatial homogeneity. Interestingly, dyneins have been found to move bidirectionally along microtubules[62], and overall mixed orientations of microtubules have been reported in dendrites[84,89–91]. A small anterograde transport bias has been reported, but its magnitude, temporal stability, and spatial homogeneity along the dendrite are now the topic of active research[13,15,50,65,67]. Furthermore, its direct consequence, a build-up of mRNAs at the dendritic tip, has not been reported. Remarkably, our symmetric transport model with experimentally reported run times and speeds leads to spatial profiles of mRNA distributions that match experimental reports of this quantity (Supplementary Figure 6).

Detailing the mathematics of the multi-state mRNA transport model. We adopted the mathematical model from chapter IV, section C of[85]. The dynamics of an mRNA population m at $t \geq 0$ comprising a diffusive subpopulation m_0 and an anterogradely (retrogradely) moving fraction m_+ (m_-) is given by the following one-dimensional hyperbolic partial differential equations:

$$\frac{\partial}{\partial t} m_+ = -v \frac{\partial}{\partial x} m_+ - \beta m_+ + \alpha m_0, \tag{1a}$$

$$\frac{\partial}{\partial t} m_- = v \frac{\partial}{\partial x} m_- - \beta m_- + \alpha m_0, \tag{1b}$$

$$\frac{\partial}{\partial t} m_0 = \beta m_+ + \beta m_- - 2\alpha m_0 + D_m \frac{\partial^2}{\partial x^2} m_0 - \lambda_m m_0. \tag{1c}$$

Here, α, β are the transition rates between the stationary and mobile states, v is the instantaneous non-negative bidirectional cargo velocity, and D_m is the diffusion constant of the diffusive subpopulation. Note that we added

a degradation term with decay rate λ_m to the diffusive population of the original model of[85]. At the boundaries of $[0, L]$, the amount of anterograde and retrograde transport has to be equal, and at $x = L$, the dendrite is closed for mRNAs. This left us with a fourth condition, so we set the concentration of mRNA at $x = 0$ to a constant value m_{const}:

$$0 = m_+(0) - m_-(0), \tag{2a}$$

$$0 = m_+(L) - m_-(L), \tag{2b}$$

$$0 = \frac{\partial}{\partial x} m_0(L), \tag{2c}$$

$$m_{const} = m_0(0) + m_+(0) + m_-(0)\,. \tag{2d}$$

The steady-state probability distribution of states is then given by

$$P_{SS}\left(m_0\right) = \frac{1}{\gamma\alpha}, \quad P_{SS}\left(m_+\right) = P_{SS}\left(m_-\right) = \frac{1}{\gamma\beta}, \tag{3}$$

with $\gamma = 1/\alpha + 1/\beta + 1/\beta$. Active transport dynamics are now determined by the motor velocity v and the switching rates α, β. We calculated α using the ratio of actively transported mRNAs θ and the fact that $\theta = P_{SS}\left(m_+\right) + P_{SS}\left(m_-\right) = 1 - P_{SS}\left(m_0\right)$. From (eq. 3) we concluded

$$\alpha = \frac{1}{\gamma P_{SS}\left(m_0\right)} = \frac{1}{\gamma\left(1-\theta\right)}, \quad \gamma = \frac{2}{\theta\beta}, \tag{4}$$

and thus

$$\alpha = \frac{\theta}{1-\theta} \cdot \frac{\beta}{2}. \tag{5}$$

Fit of ensemble diffusion constant. To quantify the increase in mRNA mobility due to active transport, we fitted a single-state diffusion model to the steady-state solution of (eq. 1). The fitted model comprises only one mRNA population with diffusion constant $\tilde{D}_m$ and degradation rate λ_m, and its steady-state distribution is given by

$$0 = \tilde{D}_m \frac{\partial^2}{\partial x^2} m - \lambda_m m, \tag{6}$$

where $\tilde{D}_m$ is the ensemble or effective diffusion constant describing the superposition of passive diffusion with intermittent active mRNA transport. With the boundary conditions

$$\frac{\partial}{\partial x} m(L) = 0, \quad m(0) = m_{const} > 0, \tag{7}$$

equation (eq. 6) possesses a simple analytical solution on the interval $[0, L]$:

$$m(x) = m_0\, exp\left(-\sqrt{\frac{\lambda_m}{\tilde{D}_m}}x\right) \frac{exp\left(2\sqrt{\frac{\lambda_m}{\tilde{D}_m}}L\right) + exp\left(2\sqrt{\frac{\lambda_m}{\tilde{D}_m}}x\right)}{exp\left(2\sqrt{\frac{\lambda_m}{\tilde{D}_m}}L\right) + 1}. \tag{8}$$

Now, we fitted (eq. 8) to the solution of (eqs. 1, 2) using a least-squares method.

Fraction of transported mRNA granules θ. In our modelling framework, we assume that, at any time, 10% of dendritic mRNA granules are in active transport. In dendrites, the fraction of active transport is generally higher than in non-neuronal cells, but the non-active population shows slower dynamics[13,48]. In rat cortical neurons, ref.[49] found that 3% of granules are motile, and this motility vanishes after microtubule depolymerisation. Because they claim that small, bidirectional movements were not tractable, they likely underestimate the share of motile granules.[50] study the dynamics of a reporter mRNA in cultured rat hippocampal neurons and find that ~2-4% of granules are motile. Moreover, ref.[51] characterise ~80% of mRNA puncta in dendrites of cultured hippocampal neurons as immobile, ~10% as oscillatory, ~5% moving ante-, and ~5% moving retrogradely.[52] also

find that most mRNA puncta is immobile in cultured hippocampal neurons. This corresponds to[53] reporting that granules are either in directed transport or oscillatory motion without net displacement. In a more recent study, ref.[13] analyse endogenous mRNA in hippocampal neurons of living mice and classify the dynamics of $\sim$48% as stationary, $\sim$30% as restricted diffusive, $\sim$12% as unrestricted diffusive, and $\sim$10% as directed. The 10% share of active transport reported by[13] is somewhat higher than others reported *in vitro*, possibly due to impaired motor protein activation in *in vitro*, which[54] has analysed for dynein.[55] find that, for one type of granules, the transported fraction decreases from around 20% in immature dendrites to 6% in mature dendrites. Even higher fractions of 40% moving granules have also been reported[56].

mRNA granule velocity v during the run phase Based on experimental reports discussed below, we employed a value of $v = 1\mu$m/s to describe the run phase of mRNA granules in the anterograde and retrograde direction. Dendrites are often idealised as one-dimensional; thus, most studies on intracellular trafficking measure velocities in the anterograde (toward the dendritic tip) and the retrograde direction (towards the soma) separately. While older studies found granule velocities in the range of 0.1-1μm/s[15,49,51,57], three studies[13,53,58] consistently report instantaneous run velocities for mRNA granules of 1-1.3μm/s in dendrites, similar to the transport velocities[48] found in non-neuronal mammalian cells. In addition, ref.[56] report a symmetric granule speed of 1μm/s for *CaMKIIα*, *β-actin*, and *PSD-95* mRNAs in dendrites. This range of velocities also matches those observed for kinesins and dyneins across different contexts[54,59–63] and motivated us to consider $v = 1\mu$m/s in our model.

Transport state exit rate β. We quantified the rate of mRNA granules leaving a transport state β by considering the experimentally observed average duration of a single mRNA granule run (the inverse of the exit rate β).[64] reported average run durations of 1-2 seconds with the corresponding velocities of 0.6-0.7 μm/s. These values match experimental reports from[65], who observed 5-6μm runs with 3μm/s speed. However, these studies excluded runs shorter than 1.5μm. With the same exclusion criterion, ref.[13] found a somewhat longer run time (5-6μm runs with 1-1.3μm/s speed). Velocity (0.8μm/s) and travelled distance (450nm) of a single dynein motor in mouse axons from[63] give a slightly shorter average run duration of 0.5s, but the same authors' data on cargoes operated by multiple motors (1.2μm/s and 5.3μm) result in longer runs of 4-5s. Similar run durations follow from data by[66] (1.5$\pm$0.7 μm/s and 6.7-9.2μm), and also match results of[58,67]. Again shorter run durations follow from the average traveled distances of dynein-dynactin mediated transport reported by[68], which are in the range of 0.5-1.5μm with speeds of up to 1.5μm/s. To consolidate these findings into a single model parameter, we used an average run length of 1 second. This value is at the lower end of reported values, but because some of the reports excluded short runs, f.e.[13,65], which can bias them toward larger average run durations, we decided to choose 1 second.

mRNA granule content. mRNAs are known to be transported as part of specialised granules[92–94]. Various reports showed that mRNA granules can contain exclusively one mRNA species[51,77,78,95] and recent studies observed that many granules may carry only one mRNA molecule[13,48,56,68,77,95]. Other experimental studies indicated the possibility that more than one mRNA could be present in a transport competent granule[96,97], e.g. 2 mRNAs (*CamKII* and *Arc*) were reported in a single granule [98]. As recent reviews indicate, the current technological advances are only beginning to tackle the molecular complexity, the sub-types, the assembly and the possible mRNA cargo inside the transport competent granules[99] such that the number of mRNAs per granule and its upper bound is not yet known but is a topic of active research[99,100]. For our model here, we thus considered one mRNA per granule throughout the main manuscript and confirmed that our main results hold when considering 2 and 10 mRNAs per granule in Supplementary Figure 18.

Experimental validation of the predicted stay durations in resting state. Having quantified all necessary model parameters, we validated the resulting mRNA mobility statistics with experimental data, particularly the predicted average resting state duration, i.e., the length of phases without transport. This duration is given by the inverse of the associated switching rate 2α (eq. 1). Using (eq. 5), we computed an average resting state duration of 9 seconds, which is in line with 5-10s resting time in diffusive states calculated by[64], and also with the observation of[58] that 71.5% of mRNAs are stationary (not transported) after 5s time. On the other hand, ref.[65] report that after 1 minute, 50% of mRNAs have not moved, implying a much higher time constant of the resting state. However, their analysis omitted relocations below 1.5μm, which would ignore most active transport runs within our model framework.

Single run duration does not affect mRNA transport cost. Motivated by the results in Figure 2, suggesting

that many protein and mRNA parameters follow a minimal energy principle, we sought to examine if this also holds for the transport model parameters, i.e., the fraction of transported mRNAs, the granule velocity, and the average single run time. To this end, we independently varied each parameter and confirmed that an increase in any of them leads to substantially more mRNA mobility (Supplementary Figure 2). Nevertheless, for the range of passive mRNA diffusion constants considered to be plausible (10^{-4} to $10^{-2}\mu$m^2/s, see 'Parameter sampling' section below), even small values for each parameter were sufficient to enhance mRNA diffusivity greatly. In contrast, more mobile cargoes ($10^{-1}\mu$m^2/s) require more powerful transport.
Now, the energy cost for mRNA transport scale with the percentage of transported mRNAs and the granule velocity but not the mean run duration (eq. 37). Energy efficiency requires these values to be reasonably small, while the potential of high run durations can be utilised for free.
Interestingly, our literature research of parameter values (see above) showed some reports of low granule velocities ($\sim 0.1\mu$m/s) but only a few suggesting intracellular speeds above 1μm/s. On the other hand, run lengths tended to be rather long (multiple seconds) than short (<1 second). Values for the transported mRNA fraction ranged from 3 to 40% without any recognisable strong tendency. Consequently, we hypothesised that the modalities of motor-mediated transport also underlie energetic constraints but felt that further research is necessary to make further conclusions.

Definition of mRNA localisation pathways of interest. This work's primary goal is to understand how energy considerations shape the localisation of mRNAs in the soma vs. dendrites. To tackle this question, we must first precisely define localisation pathways for mRNA whose energetic optimality we can subsequently compare. There were three straightforward candidates: 1) somatic mRNA, 2) dendritic mRNA without transport, and 3) dendritic mRNA with transport. Here, we show that 1) and 2) lead to similar mRNA and protein distributions and energy costs. For these reasons, we excluded option 2) from our considerations, restricting the number of localisation pathways of interest to two: somatic mRNA and dendritic mRNA using active transport.
Our transport model framework applied to a biologically plausible range of mRNA parameters revealed that microtubular transport of mRNA granules is, in fact, necessary to shift mRNAs from very proximal regions into dendrites (Supplementary Figure 6). In other words, we observed similar mRNA distributions if mRNAs are either retained in the soma or put into the dendrite without motor-mediated trafficking.

Obtaining spatial distributions of mRNAs and proteins

Model equations. With the model for active mRNA transport described above and the fitted ensemble mRNA diffusion constant $\tilde{D}_m$, we could then write down the equations covering mRNA and protein dynamics in a linear dendrite of length L. In the following, variables indexed with m (p) always represent mRNA (protein) properties, and m and p represent the dendritic population of mRNA and protein. Following previous works[11,101], we assumed that mRNA and protein synthesis, mobility, and degradation are the major influences shaping the global intracellular distribution. First, we covered protein synthesis dynamics in a single parameter, the translation rate τ. We assumed that it is similar for both somatic and dendritic mRNAs of the same species[24], wherefore we applied the same translation rate throughout soma and dendrite. mRNA and protein mobility is represented by their respective diffusion constants $\tilde{D}_m, D_p$. Protein motion within spines was excluded from our considerations because it does not directly influence the macroscopic spread of proteins along the dendrite. Protein and mRNA degradation were modeled by decay rates $\lambda_m \lambda_p$, which were defined based on molecule half-lives $T^{1/2}$ via $\lambda = ln(2)/T^{1/2}$. Likewise, spine proteins are degraded[102,103], and we applied similar degradation rates in spines and dendrites. We extended previous global mRNA and protein distribution models by introducing a dynamic protein exchange between the dendritic shaft and spines. Following the propositions of[104], we introduced a population of proteins in spines p_{spine}. With ρ, we denoted the spatial spine density. Dendritic proteins are incorporated into spines with an uptake rate u_p and leave spines with an exit rate ν_p. Again following[104], we assumed that the uptake does not linearly depend on u_p but that a spine's content is thresholded by a maximal protein capacity η_p. For a spine at location $x \in [0, L]$ we obtained protein uptake and exit of

$$\underbrace{u_p p(x) \left(1 - \frac{p_{\text{spine}}(x)}{\rho \eta_p}\right)}_{uptake} - \underbrace{\nu_p p_{\text{spine}}(x)}_{exit}. \tag{9}$$

Note that the uptake threshold in[104] is given by $(\rho\eta_p - p_{\text{spine}}(x))$, meaning that the uptake of a spine with a given supply ratio $\phi = \frac{p_{\text{spine}}}{\rho\eta_p}$ depends linearly on its capacity η_p:

$$u_p p\left(\rho\eta_p - p_{\text{spine}}\right) = u_p p\left(\rho\eta_p - \phi\rho\eta_p\right) \sim \eta_p. \tag{10}$$

Hence, proteins with more copies per spine would experience different dynamics at the same level of spine supply ϕ. Still, in this work, we aimed to compare among protein species given a similar supply ratio ϕ. We eliminated this difference by normalising with $\rho\eta_p$, leading to (eq. 9). Finally, molecule mobility $(\tilde{D}_m, D_p)$, degradation (λ_m, λ_p), protein synthesis (τ), and spine dynamics $(u_p, \nu_p, \eta_p, \rho)$ define the mathematical model for mRNA and protein distribution in dendrite and spines via

$$\frac{\partial}{\partial t}m = \tilde{D}_m \frac{\partial^2}{\partial x^2}m - \lambda_m m, \tag{11a}$$

$$\frac{\partial}{\partial t}p = D_p \frac{\partial^2}{\partial x^2}p - \lambda_p p + \tau m - u_p p\left(1 - \frac{p_{\text{spine}}}{\rho\eta_p}\right) + \nu_p p_{\text{spine}}, \tag{11b}$$

$$\frac{\partial}{\partial t}p_{\text{spine}} = -\lambda_p p_{\text{spine}} + u_p p\left(1 - \frac{p_{\text{spine}}}{\rho\eta_p}\right) - \nu_p p_{\text{spine}}, \tag{11c}$$

with $m, p, p_{\text{spine}} \in \mathcal{C}^2([0, L], \mathbb{R})$, and $D_m, \lambda_m, D_p, \lambda_p, u_p, \nu_p, \eta_p, \rho, \tau \in \mathbb{R}_{>0}$. The parameters used in (eq. 11) are summarised in Supplementary Table 3. Next, we focused on the steady-state $\frac{\partial}{\partial t} = 0$ of (eq. 11):

$$0 = \tilde{D}_m \frac{\partial^2}{\partial x^2}m - \lambda_m m, \tag{12a}$$

$$0 = D_p \frac{\partial^2}{\partial x^2}p - \lambda_p p + \tau m - u_p p\left(1 - \frac{p_{\text{spine}}}{\rho\eta_p}\right) + \nu_p p_{\text{spine}}, \tag{12b}$$

$$0 = -\lambda_p p_{\text{spine}} + u_p p\left(1 - \frac{p_{\text{spine}}}{\rho\eta_p}\right) - \nu_p p_{\text{spine}}, \tag{12c}$$

Equations (eqs. 12b, 12c) allowed us to write down a formula for p_{spine} directly:

$$p_{\text{spine}} = \frac{u_p p}{\left(\frac{u_p}{\rho\eta_p}\right)p + \nu_p + \lambda_p} = \frac{\pi_p p}{\left(\frac{\pi_p}{\rho\eta_p}\right)p + 1}, \tag{13}$$

where $\pi_p = \frac{u_p}{\nu_p + \lambda_p}$. We called π_p the *permeability* of the spine. With (eq. 13), we reduced (eq. 12) to

$$0 = \tilde{D}_m \frac{\partial^2}{\partial x^2}m - \lambda_m m, \tag{14a}$$

$$0 = D_p \frac{\partial^2}{\partial x^2}p - \lambda_p p - \lambda_p \frac{\pi_p p}{\left(\frac{\pi_p}{\rho\eta_p}\right)p + 1} + \tau m. \tag{14b}$$

Boundary conditions. First, we assumed that the dendrite is closed at $x = L$ for mRNAs and proteins, i.e.,

$$\begin{aligned} \frac{\partial}{\partial x}m(L) &= 0, \\ \frac{\partial}{\partial x}p(L) &= 0. \end{aligned} \tag{15}$$

Now, proteins can be either translated by somatic mRNAs $m_{\text{soma}} = m_{\text{soma}}\delta(x)$ or mRNAs in the dendrite $m = m(x)$. For mRNAs, we introduced the somatic retention ratio $r_{\text{soma}} \in [0, 1]$ describing the fraction of mRNAs retained in the soma:

$$r_{\text{soma}} := \frac{m_{\text{soma}}}{m_{\text{soma}} + \int_0^L m(x)\,dx}. \tag{16}$$

We named the case $r_{\text{soma}} = 1$ somatic translation. If not stated differently, the term dendritic translation, in contrast, refers to $r_{\text{soma}} = 0$ and $\theta_m > 0$, i.e., all mRNAs are in the dendrite and are to some degree actively

transported. The translation of somatic mRNAs (which are only present if $r_{\text{soma}} > 0$) will lead to a protein influx into the dendrite at $x = 0$ given by

$$D_p \frac{\partial}{\partial x} p(0) = -\tau m_{\text{soma}}. \tag{17}$$

To derive a condition for mRNAs at the soma, we solved the integral $\int_0^L m(x)\,dx$:

$$\int_0^L m(x)\,dx \overset{(eq.\ 12a)}{=} \int_0^L \frac{\tilde{D}_m}{\lambda_m} \frac{\partial^2}{\partial x^2} m(x)\,dx = \frac{\tilde{D}_m}{\lambda_m} \left(\frac{\partial}{\partial x} m(L) - \frac{\partial}{\partial x} m(0) \right) \overset{(eq.\ 15)}{=} -\frac{\tilde{D}_m}{\lambda_m} \frac{\partial}{\partial x} m(0). \tag{18}$$

Then we could rewrite (eq. 16) in the case $r_{\text{soma}} < 1$:

$$m_{\text{soma}} = \frac{r_{\text{soma}}}{1 - r_{\text{soma}}} \int_0^L m(x)\,dx = -\frac{r_{\text{soma}}}{1 - r_{\text{soma}}} \frac{\tilde{D}_m}{\lambda_m} \frac{\partial}{\partial x} m(0), \tag{19}$$

which we could in turn insert in (eq. 17) to obtain the next boundary condition at $x = 0$ for $r_{\text{soma}} < 1$:

$$D_p \frac{\partial}{\partial x} p(0) = \tau \frac{r_{\text{soma}}}{1 - r_{\text{soma}}} \frac{\tilde{D}_m}{\lambda_m} \frac{\partial}{\partial x} m(0). \tag{20}$$

Note that for $r_{\text{soma}} = 0$, this reduces to

$$D_p \frac{\partial}{\partial x} p(0) = 0. \tag{21}$$

If on the other hand $r_{\text{soma}} = 1$, there will be no dendritic mRNA, i.e.,

$$0 = \begin{cases} D_p \frac{\partial}{\partial x} p(0) - \frac{r_{\text{soma}}}{1 - r_{\text{soma}}} \frac{\tau \tilde{D}_m}{\lambda_m} \frac{\partial}{\partial x} m(0), & r_{\text{soma}} < 1, \\ \frac{\partial}{\partial x} m(0), & r_{\text{soma}} = 1. \end{cases} \tag{22}$$

Finally, we assumed that all spines are supplied with a certain minimal ratio $\phi \in [0, 1)$ of their maximal protein capacity $\rho\eta_p$ to remain functional. In particular, the last spine at $x = L$ has to receive its appropriate amount of protein $\phi\rho\eta_p$. Using (eq. 13), we formalised this as

$$p_{\text{spine}}(L) = \phi\rho\eta \iff p(L) = \frac{1}{\pi_p} \frac{\phi}{(1 - \phi)} \rho\eta_p. \tag{23}$$

Now, consolidating (eqs. 15, 22, 23) we obtained as boundary conditions for (eq. 14):

$$0 = \frac{\partial}{\partial x} m(L), \tag{24a}$$

$$0 = \frac{\partial}{\partial x} p(L), \tag{24b}$$

$$0 = \begin{cases} D_p \frac{\partial}{\partial x} p(0) - \frac{r_{\text{soma}}}{1 - r_{\text{soma}}} \frac{\tau \tilde{D}_m}{\lambda_m} \frac{\partial}{\partial x} m(0), & r_{\text{soma}} < 1, \\ \frac{\partial}{\partial x} m(0), & r_{\text{soma}} = 1, \end{cases} \tag{24c}$$

$$0 = p(L) - \frac{1}{\pi_p} \frac{\phi}{(1 - \phi)} \rho\eta_p \tag{24d}$$

Numerical solution. To numerically solve our model given by (eqs. 14, 24), we used the MATLAB solver *bvp5c* (https://www.mathworks.com/help/matlab/ref/bvp5c.html): *bvp5c* is a finite difference code that implements the four-stage Lobatto IIIa formula, which is a collocation formula. The collocation polynomial provides a C^1-continuous solution that is fifth-order accurate uniformly in $[a, b]$. The formula is implemented as an implicit Runge-Kutta formula.

Translation rate τ. Proteins are synthesised by ribosomes translating the nucleotide sequence of mRNAs into a chain of amino acids constituting a protein[105]. First, it has to be noted that only a fraction of mRNAs is actively

translated, and this ratio seems to be quite heterogeneous among mRNAs[106], cell types, temporally[82,107], and even spatially in neurites[107]. This ratio can be as high as 70% for a reporter mRNA fixed syntheticly to the plasma membrane[82]. In contrast, the same group reported that ∼50% of short and ∼20% of long isoform transcripts of the same mRNA were translated. In direct comparison, long isoform translation was 40-fold suppressed because only a minority of long isoforms were translated strongly. In dendrites, ref.[107] find that within 30μm from the soma, the ratio is 0.4, the same as in glia. However, it decreases to 0.1 after 100μm from the soma. Additionally, several experiments suggest that translation is repressed during dendritic transport[79,83,108–110], arguing for a relatively low ratio of translated mRNA in dendrites. This aligns with the fact that the reported ratios of translated mRNAs (≤70%) are smaller than the fraction of diffusive mRNAs in our model (90%).
The second step in defining translation dynamics is the protein synthesis rate of a presently translated mRNA. One mRNA is translated from multiple ribosomes simultaneously, forming a polyribosome.[111] argue that the limiting step of translation dynamics is its initiation rather than its elongation. Initiation rates can be either measured directly or derived from protein elongation speed and inter-ribosomal distance measurements, which vary from 200-400 nucleotides[82]. Finally, measured initiation (hence protein synthesis) rates are 1.3-2.1min^{-1}[107], ∼2min^{-1}[112], 2.4min^{-1}[113], 1.4-3.6min^{-1}[82]. Furthermore, ref.[107] analysed temporal dynamics of translation in dendrites and found that overall dynamics are similar among processive translation and mRNAs experiencing translational bursts. Combining the data on the fraction of translated mRNA in dendrites and their synthesis speed (Supplementary Table 4), we concluded to use a dendritic translation rate of 0.01 proteins per second and mRNA. This value agrees with the median translation rate per mRNA found in mouse fibroblasts[6]. Following[24], we used the same translation rate per mRNA in somata and dendrites.
To give an intuition of τ, one can calculate the number of proteins synthesised during an average mRNA lifetime. With mRNA half-lives in the 2 to 20-hour range, this results in 70-700 produced proteins for a typical mRNA lifespan.

Spine density ρ. Many studies report on the dendritic spine density, but their findings are rather heterogeneous.[114] find a 0.19-0.26 spines per μm dendrite in various Brodmann areas of human cortex.[115] report larger densities in macaque monkeys with 0.5-2μm^{-1}, who also observe significant differences among different cortices similar to[116], finding even higher values of 1-2μm^{-1}. Also[117] find differences among cortices and several mammalian species, reporting 0.7-3.2 spines per μm maximal spine density. These values align with the 1-2μm^{-1} observed by[118], who, in contrast, do not find significant differences between mouse cortices.[119] analyse CA1 hippocampal neurons of mice and find 2 spines per μm, similar to[120]. These differences might be due to differences in cell preparation because, for example, rat hippocampal neurons have, on average, much less (0.2μm^{-1}) dendritic spines when in dispersion culture than in organotypic slice culture (1.5μm^{-1})[121].[122] find differences between adult and juvenile rat cells with spine densities within 1.4-3.2μm^{-1}. Even the estrus cycle influences the spine density in female rats[123]. For our model, we applied a constant spine density of 1μm^{-1} along the dendrite to keep its predictions as generic as possible.

Spine supply ratio ϕ and spine dynamics u_p, ν_p. For the spine supply ratio ϕ, we chose 95%, leading to an almost constant amount of proteins in spines (between 0.95η_p and η_p per spine) along the dendrite. With this choice for ϕ, we found that a permeability π_p of 1000 is well suited to reproduce three independent experimental observations: 1) The protein abundance in neurons as reported by us[5], see Figure 4; 2) the dynamic integration of somatic proteins in dendritic spines[44], see Figure 5; 3) protein spine-to-dendrite-ratios of 2-15 species, computed from our imaging data[5]. Note that in our modelling framework, π_p was defined based on the choice of ϕ. To simulate spatiotemporal spine dynamics (Figure 5), we additionally needed a value for the rate of proteins leaving spines ν_p. The relatively strong permeability π_p implies that the uptake rate u_p is way higher than the exit rate: $u_p \approx \pi_p \nu_p = 1000\nu_p$. In other words, ν_p dictates the rate of protein replacement in spines, and the latter can be estimated from the literature. Although the spine neck constitutes a diffusional barrier for presumably non-interacting and small molecules like *GFP* or polysaccharides, their replacement in spines is fast with time constants on the order of milliseconds to seconds[124–126]. This corresponds to results obtained by simulating diffusive trapping in realistic spine geometries[127]. Still, resting times in spines of endogenous proteins are not only defined by mere trapping but are also affected by physical hindrances[128] as well as interactions with and binding to partners within spines. This fact is well reflected in a study modelling *CaMKII* protein dynamics[129]. They show that proteins are trapped ∼10min in spines when considering spine geometry and binding, while retention times drop to the order of seconds when considering either geometry or binding alone. Further diffusion simulations in spine-inspired geometries also suggest time constants of a few minutes for cytosolic and membrane proteins[130,131]. These values match

experimental observations: While[132] report time constants of a few seconds for *CaMKII* proteins leaving spines, refs.[133,134] find that *CaMKII*α and actin proteins departing spines can be represented by two populations with time constants $\sim$1min and $\sim$20min. Furthermore, ref.[124] report time constants of $\sim$4(2)min for *AMPAR* in mushroom (stubby) spines but only for the mobile population. In both cases, the mobile fraction is $\sim$50%, in line with[135]. In addition, ref.[125] report time constants of a few minutes for mobile *CaMKII*, *GluR1*, *PSD95*, and *NR1* with immobile fractions of 30-80%, both varying among protein species. In contrast to experimental studies, which often report retention times in spines for 'fast' and 'slow' or 'mobile' proteins, we here treated the total protein population as a whole. Therefore, we accounted for this by using a time constant of 10min and hence $\nu_p = \ln(2)/10min$, covering mobile, immobile, fast, and slow proteins.

Discrete spines

Throughout this work, we formalised the proteins in the dendritic shaft and spines as continuous distributions p and p_{spine}. While this is natural for the dendritic shaft, spines, on the contrary, are discrete protrusions, which is indeed reflected in other studies modelling dendrite-spine interactions, e.g., ref.[104]. We could also modify our model to simulate spines at discrete locations (Supplementary Figure 13). Here, we show that, given a reasonably high spine density ρ, the continuous and discrete versions of the model lead to similar outputs for equidistant spine locations. To this end, we began by formulating the discretised version of the steady-state model (eq. 14). On a linear dendrite of length L we chose spine locations $\{s_i\}_{i=1,\dots,n}$, $0 < s_1 < \cdots < s_n < L$. For simplicity, we used $s_0 := 0$ and $s_{n+1} := L$ (but there are no spines at $\{0, L\}$). The spine density ρ and the dendrite length define the spine number: $n = \rho \times L$. We called the spine locations $\{s_i\}_{i=1,\dots,n}$ equidistant, if $|s_i - s_{i-1}| = L/(n+1)$ for all $i = 1, \dots, n+1$. We then defined dendritic mRNA and protein populations m_i, p_i within each interval (s_{i-1}, s_i), $i = 1, \dots, n+1$ and applied the continuous model (eq. 14) without spine-dendrite interactions for proteins:

$$0 = \tilde{D}_m \frac{\partial^2}{\partial x^2} m_i - \lambda_m m_i, \tag{25a}$$

$$0 = D_p \frac{\partial^2}{\partial x^2} p_i - \lambda_p p_i + \tau m_i. \tag{25b}$$

Interactions between dendrite and spine are in this discrete version limited to the spine locations $s_1, \dots, s_n$. We formalised them as conditions at each spine s_i, $i = 1, \dots, n$:

$$m_{i+1}(s_i) = m_i(s_i), \tag{26a}$$

$$p_{i+1}(s_i) = p_i(s_i), \tag{26b}$$

$$\frac{\partial}{\partial x} m_{i+1}(s_i) = \frac{\partial}{\partial x} m_i(s_i), \tag{26c}$$

$$\frac{\partial}{\partial x} p_{i+1}(s_i) = \frac{\partial}{\partial x} p_i(s_i) - \overbrace{\lambda_p \frac{\pi_p p_i(s_i)}{\left(\frac{\pi_p}{\eta_p}\right) p_i(s_i) + 1}}^{\text{spine } uptake\ (eq.\ 14b)}. \tag{26d}$$

Here, we directly applied the reduced version of the full spine-dendrite interaction (eq. 9) and hence obtained the protein population in the spine s_i:

$$p_{\text{spine},i} = \frac{\pi_p p_i}{\left(\frac{\pi_p}{\eta_p}\right) p_i + 1}. \tag{27}$$

It remained to transfer the boundary conditions from the continuous (eq. 24) to the discrete case. The first three conditions could be transferred directly:

$$0 = \frac{\partial}{\partial x} m_{n+1}(s_{n+1}), \tag{28a}$$

$$0 = \frac{\partial}{\partial x} p_{n+1}(s_{n+1}), \tag{28b}$$

$$0 = \begin{cases} D_p \frac{\partial}{\partial x} p_1(s_0) - \frac{r_{\text{soma}}}{1 - r_{\text{soma}}} \frac{\tau \tilde{D}_m}{\lambda_m} \frac{\partial}{\partial x} m_1(s_0), & r_{\text{soma}} < 1, \\ \frac{\partial}{\partial x} m_1(s_0), & r_{\text{soma}} = 1. \end{cases} \tag{28c}$$

Finally, the minimal supply condition (eq. 23) applied to the last spine s_n, not the dendritic tip $s_{n+1} = L$:

$$p_{\text{spine},n} = \phi\eta \iff p_n(s_n) = \frac{1}{\pi_p} \frac{\phi}{(1 - \phi)} \eta_p, \tag{29}$$

thus leading to the last boundary condition

$$p_n(s_n) = \frac{1}{\pi_p} \frac{\phi}{(1 - \phi)} \eta_p. \tag{30}$$

In the end, we defined $4n + 4 = 4(n + 1)$ conditions for the same number of equations.

Sampling of synthetic protein species

We sampled values from published databases covering thousands of mRNA and protein species to analyse the effect of seven mRNA and protein parameters on the energy efficiency of dendritic mRNA localisation. For each of the seven parameters of interest, we chose three values (low, medium, and high, see Supplementary Table 6 for an overview of parameter ranges). With three values, we could cover a broad range of parameter values, which was necessary to detect the macroscopic statistical effects of each parameter on the energy cost. The low, medium and high values were picked by hand to best represent the mean and the 2-3 σ interval left and right of the mean across published data. For example, we sampled copy numbers per spine from a single published source[5] but we located five databases with protein half-lives in neurons, covering multiple experimental paradigms, reporting different sample numbers and data analysis pipelines[1–4]. Here, we chose our three parameter values to account for all available distributions (Supplementary Figure 1).
First, we found that half-lives of proteins *in vivo*[1,2] are higher than those *in vitro*[3,4] (Supplementary Figure 1A). We sampled 2, 8, and 20 days to account for both experimental paradigms.
For the protein size (in amino acids per protein), we took 100, 500, and 2000 as parameter values, relying on the database of[2] (Supplementary Figure 1B).
Next, we collected more than 30 protein diffusion constants D_p in neurites (Supplementary Figure 1D, Supplementary Table 1). Around 50% of entries were similar to those gathered by[101]. From the resulting distribution (Supplementary Figure 1D) we sampled the parameter values $10^{-2.5}$, $10^{-1.5}$, $10^{-0.5}$ μm^2/s for the protein diffusivity D_p.
We[5] provide data on the number of proteins per spine, represented by η_p in this study, for more than 100 protein species. We used 10, 200, and 5000 proteins per spine in our parameter space (Supplementary Figure 1C).
In comparison to protein, reports on mRNA half-lives were relatively consistent. Because mRNA half-lives are typically measured within short intervals and interpolation to larger intervals can be error-prone, ref.[9] dropped values above 24 hours, whereas[8] set values larger than 24 hours to 24 hours. Following this notion, we picked 20 hours as the highest parameter value, taking the risk of ignoring longer-lived mRNAs (Supplementary Figure 1E). The two smaller values we chose were 2 and 8 hours.
For each species, we sampled nucleotide counts per mRNA such that 1) each species could have multiple transcripts of different lengths, and 2) longer coding sequences would offer more options for introns that are not present in mature transcripts but must be transcribed and thus are a relevant cost factor. To this end, we introduced the ratio of non-coding to coding nucleotides, where non-coding nucleotides cover untranslated regions and introns. We chose three ratios for each protein length (100, 500, 2000 amino acids): 0, 3, and 12 non-coding nucleotides per 3-nucleotide codon within the coding sequence. With these ratios, we covered mRNAs transcribed without

non-coding nucleotides up to those with multiple introns containing up to thousands of nucleotides each[74–76].

In our model, (passive) mRNA diffusion constants represent cytosolic movements without the contribution of microtubule transport. Studies reporting a single diffusion constant for the whole population often do not differentiate between mobility states; thus, their calculated diffusion constants presumably cover the total intracellular motion. On the contrary, we wanted to determine the diffusivity of mRNAs not being in a transport state. Because mRNAs with typically $>$100nm diameter[51,77–80] are much bigger than proteins, we assumed that the range of protein diffusion constants constitutes an upper bound for passive mRNA mobility. Moreover, a modelling study emphasised the physical hindrance the cytoskeleton imposes on molecules with diameters as small as 8nm[81], suggesting similar or even more substantial diffusion restrictions on objects as large as mRNA granules. A further lower bound for mRNA mobility is given by mRNAs classified as 'immobile', which often constituted most of the non-transported population[10,15,49,51,52,55]. Some fraction of mRNAs frequently labelled as immobile is most likely bound to another intracellular structure, for example, the plasma membrane, in which case[82] determined an mRNA diffusion constant of 0.00106μm^2/s. This value was surprisingly similar to the lowest values reported for the ensemble mRNA diffusion constants (i.e., including transport) in experimental[13] and modelling studies[64], arguing for the presence of even less mobile mRNAs. Finally, the fact that a significant share of mRNAs seems to be masked against standard imaging protocols[10] underlined the high degree of uncertainty the reported diffusion constants are subject to. Therefore, we chose the rather broad range of 10^{-4}, 10^{-3}, 10^{-2} μm^2/s for the passive mRNa diffusion constant. A posteriori, we found that, within this range of values, the mRNA diffusion constant had no considerable influence on the mRNA distribution in the presence of active mRNA transport (Supplementary Figures 6, 2).

Calculating the energy cost

Introduction. This work's overarching goal was to quantify the energy cost of protein localisation. Therefore, we considered only energy-consuming processes specifically involved in protein localisation for our calculations and excluded the cost for 'infrastructure', e.g., mitochondria. Their synthesis and transport will lead to significant energetic expenditures, but mitochondria in dendrites serve as energy providers for various processes like voltage transduction[136]. The steps we considered to be the major cost factors for protein localisation in dendrites were mRNA and protein synthesis, degradation, and active transport along microtubules.

Transcription cost. First of all, mRNAs have to be synthesised from DNA. For the associated cost, we followed[75]. They found that the cost to synthesise a single mRNA is predominantly given by nucleotide synthesis, chain elongation, and, in eukaryotes, posttranscriptional modifications. Degradation of transcripts does not lead to additional costs, and activation and initiation of transcription, splicing, 5' capping, and mRNA export are only minor cost factors. Because nucleotides can be recycled efficiently[75], we assumed that synthesising new nucleotides plays a minor role in mature neurons. Therefore, nucleotide chain elongation is the critical cost factor for transcribing a single mRNA. It depends on the cost to add a single nucleotide C_{nt} and the number of nucleotides per mRNA N_{nt}:

$$C_{\text{mRNA}} = C_{\text{nt}} N_{\text{nt}} \,. \tag{31}$$

Chain elongation takes 2ATP per nucleotide[75,137], and posttranscriptional modifications in eukaryotes lead to an additional 0.17ATP per nucleotide[75]. Here, we used the value for eukaryotes. We used the parameters obtained by the abovementioned sampling strategy for the mRNA length. Overall transcription costs were then given by the cost per single mRNA C_{mRNA} and the number of synthesised mRNAs. Because we analysed the baseline cost, mRNA transcription equalled mRNA degradation. In summary, we obtained

$$\begin{aligned} C_{\text{transcr}} &= C_{\text{nt}}\, N_{\text{nt}}\, \underbrace{\lambda_m \left(m_{\text{soma}} + \int_0^L m(x)\, dx \right)}_{\text{total mRNA decay}} \\ &= 2.17\text{ATP}\, N_{\text{nt}}\, \lambda_m \left(m_{\text{soma}} + \int_0^L m(x)\, dx \right) . \end{aligned} \tag{32}$$

Translation and protein degradation cost. Fully transcribed mRNAs are translated to proteins by ribosomes. We did again follow[75] for the quantification of related costs. They concluded that the costs for protein synthesis are

predominantly given by amino acid synthesis, chain elongation, and degradation. In contrast, they considered the cost for translation initiation and termination, posttranslational modifications, ribosomal proofreading, and protein folding negligible. Because amino acids can be recycled, we regarded the cost for amino acid synthesis in mature neurons to be small compared to the ongoing cost for chain elongation, which amounts to 4ATP per elongation step[75,137,138]. In contrast to mRNAs, protein degradation does consume energy, that is, 1ATP per amino acid[75]. For one protein comprising N_{aa} amino acids, the cost for synthesis and degradation per amino acid C_{aa} hence sum to

$$\begin{aligned} C_{\text{protein}} &= C_{\text{aa}} N_{\text{aa}} \\ &= 5\text{ATP} \times N_{\text{aa}} \,. \end{aligned} \tag{33}$$

In a steady state, the number of synthesised and degraded proteins is the same and equals the total number of mRNAs times their translation rate τ, leading to the protein-related cost of

$$\begin{aligned} C_{\text{transl}} &= C_{\text{aa}}\, N_{\text{aa}}\, \underbrace{\tau \left(m_{\text{soma}} + \int_0^L m(x)\, dx \right)}_{\text{total synthesised protein}} \\ &= 5\text{ATP}\, N_{\text{aa}}\, \tau \left(m_{\text{soma}} + \int_0^L m(x)\, dx \right) . \end{aligned} \tag{34}$$

For simplicity, we subsumed the protein-related cost (eq. 34) under 'translation cost'.
In line with[75], we moreover confirmed that for all synthetic protein species used in this work, translation costs far exceed transcription costs (Supplementary Figure 5B).

Transport cost. Dendritic trafficking of mRNA granules and protein vesicles comprises various mechanisms. Particles move through the viscous cytosol[139] by diffusion, hindered by intracellular crowding and the cytoskeleton, i.e., actin filaments[81], intermediate filaments[140,141] and microtubules[48]. The cytoskeleton is by no means a passive, rigid network. Filaments are constantly 'wiggled' by power strokes of non-processive motor proteins like *MyosinII*[142], leading to enhanced diffusivity[143,144]. We did not include the activity of non-processive motors in our energy considerations because they enhance general cytosolic motility rather than move a specific cargo.
In contrast, processive motors like kinesins and dyneins consume ATP directly attributable to cargo. Both kinesins and dyneins move in consecutive steps of 8nm[60,140,145,146]. Each step leads to the consumption of 1ATP[147–150]. mRNA granules move with $\sim 1\mu$m/s in both directions (see the transport model section above), being in the range of reported single motor speeds[54,59,60,63,151]. Yet, how many dyneins and kinesins are attached to one cargo is not fully understood. For example, ref.[60] reported that up to 11 motor proteins could simultaneously pull cargo in one direction. However, only the net number of motor protein steps executed per time determines the net instantaneous cargo velocity. Consequently, we concluded that in both directions, either one or multiple motors are pulling with a resulting speed of 1μm/s, consuming 1ATP per 8nm. Moving one cargo, i.e., mRNA granule, along microtubules with 1μm/s hence leads to a cost of

$$C_{\text{cargo}} = \frac{1\text{ATP}}{8nm} \times 1\frac{\mu m}{s} = 125\frac{\text{ATP}}{s} \,. \tag{35}$$

The overall cost depends on how many cargos N_{cargo} are transported. With the fraction of transported mRNAs θ_m, we obtained

$$N_{\text{cargo}} = \theta_m \underbrace{\int_0^L m(x)\, dx}_{\text{total dendritic mRNA}} \,, \tag{36}$$

and finally for the transport cost

$$\begin{aligned} C_{\text{transp}} &= C_{\text{cargo}}\, N_{\text{cargo}} \\ &= 125\frac{\text{ATP}}{s}\, \theta_m \int_0^L m(x)\, dx. \end{aligned} \tag{37}$$

Our approach dismissed the cost of transport granule formation and (de-)attachment to (from) microtubules. We expected these costs to be much lower than the 125ATP for an average cargo run of 1μm. Furthermore, we did not consider the cost of providing sufficient motor proteins, mRNA granule components, microtubules, and local

ATP pools. Because they all play a role in various intracellular processes, it is hard to quantify how much cost can be assigned specifically to mRNA transport. Moreover, we disregarded the potential cost of transporting mRNA and protein components and debris back to the place of mRNA or protein synthesis.

Total cost. Using (eq. 19), we added (eqs. 32, 34, 37) to finally obtain for the total cost

$$\begin{aligned} C_{\text{total}} &= C_{\text{transcr}} + C_{\text{transl}} + C_{\text{transp}}\delta_{\text{transp}} \\ &= \left(2.17\text{ATP}\, N_{\text{nt}}\, \lambda_m + 5\text{ATP}\, N_{\text{aa}}\, \tau + 125\frac{\text{ATP}}{s}\,\theta_m\, (1 - r_{\text{soma}})\,\delta_{\text{transp}}\right)\left(m_{\text{soma}} + \int_0^L m(x)\,dx\right), \end{aligned} \tag{38}$$

where $\delta_{\text{transp}} = 1$ if dendritic mRNAs use active transport and 0 if they do not.

Simulating the spread and synaptic integration of somatic proteins

We wanted to test if our model could reproduce the dynamic incorporation of somatically photoactivated *ProSAP2* proteins into single postsynaptic density (PSD) as reported in[44]. *ProSAP2* is another name for the postsynaptic protein *Shank3* (SH3 and multiple ankyrin repeat domains protein 3). Here, we considered a linear dendrite model with two protein populations, the non-photoactivated proteins p and the photoactivated proteins $\tilde{p}$. Following experimental reports[9,16,24,27,152], we considered a somatic and a dendritic *Shank3* mRNA population m_{soma} and m.

***Shank3* parameters.** In cultured rat primary hippocampal cells[3] measured a *Shank3* lifetime of 5.7d, while[2] observed much longer lifetimes in neuronal mouse tissue *in vivo*: 19.57d in cortex homogenate, 19.55d (12.87d) in cortex (cerebellum) synaptosome, and 20.04d (12.61d) in cortical (cerebellar) synaptic vesicles. Even longer half-lives were reported by[4] in mouse primary cortical neurons *in vitro*. Two replicates showed lifetimes of 27.18d and 32.97d. We, therefore, used an average lifetime of 20d for *Shank3*. As *Shank3* diffusion constant, we employed 0.9μm^2/s reported by[44]. We could not find reports of actively transported *Shank3* protein in dendrites. For the number of *Shank3* copies per spine, we[5] found 53.2±8.6 (105±17) per PSD of a mushroom (stubby) spine, slightly more than the 39 copies per PSD estimated by[153], using the established number of 300 *PSD-95* proteins per PSD[154] and the ratio of *Shank3* to *PSD-95*. With a similar approach, ref.[155] calculated 50 *Shank3* copies per PSD. A lower bound for the protein copy number per spine can be derived from *Shank1* protein abundance, which is consistently reported to be more sparse in PSDs than the related *Shank3*[153,155]. The *Shank1* to *PSD-95* ratio of 0.13 from[156] combined with an estimated 300 *PSD-95* per PSD gives a lower bound of 39 for *Shank3*, finally leading us to the conclusion that a *Shank3* count per PSD of 50 would be reasonable. *Shank3* mRNA is known to be present in dendrites, but the reported ratios of dendritic versus somatic *Shank3* mRNAs vary among studies and experimental paradigms. Published values are 0.2[9], 0.49[16], 2.12[152], 2.14[9], 2.54[152], 3.00[24], and 3.55[27]. To account for the broad range of values, we assumed the ratio of dendritic to somatic *Shank3* mRNAs to be 1; in other words, 50% of mRNAs would be retained in the soma. *Shank3* mRNA half-lives are reported as 4.68h[8], 12.68h[9], and 18.2h[152], and we hence decided to use the intermediate half-life of 12.68h. To our knowledge, the *Shank3* mRNA diffusion constant has not yet been explicitly measured, so we employed 0.001μm^2/s as the intermediate mRNA diffusivity in our parameter space sampling.[9] found *Shank3* mRNAs hundreds of microns away from the soma, which is impossible with our mRNA half-life values and passive diffusion constant (Supplementary Figure 6). Therefore, we concluded that *Shank3* mRNAs do rely on active transport. The subsequent simulations were then performed on a 750μm long linear dendrite. In summary, our parameters read:

$$D_p = 0.9\mu m^2/s, \quad \lambda_p = \frac{\ln(2)}{20d}, \quad D_m = 0.001\mu m^2/s, \quad \lambda_p = \frac{\ln(2)}{12.68h}, \quad \eta_p = 50, \quad r_{\text{soma}} = 0.5. \tag{39}$$

Baseline distribution. At $t = 0$, i.e., before photoactivation, the steady state is defined only in terms of p and the corresponding dendritic mRNA m. The governing equations are the already established equations (eq. 14):

$$0 = D_m \frac{\partial^2}{\partial x^2} m - \lambda_m m, \tag{40a}$$

$$0 = D_p \frac{\partial^2}{\partial x^2} p - \lambda_p p - \lambda_p \frac{\pi_p p}{\left(\frac{\pi_p}{\rho \eta_p}\right) p + 1} + \tau m, \tag{40b}$$

with the boundary conditions (eq. 24):

$$0 = \frac{\partial}{\partial x} p(L), \tag{41a}$$

$$0 = D_p \frac{\partial}{\partial x} p(0) - \frac{r_{\text{soma}}}{1 - r_{\text{soma}}} \frac{\tau D_m}{\lambda_m} \frac{\partial}{\partial x} m(0) \tag{41b}$$

$$0 = p(L) - \frac{1}{\pi_p} \frac{\phi}{(1-\phi)} \rho \eta_p, \tag{41c}$$

$$0 = \frac{\partial}{\partial x} m(L). \tag{41d}$$

The chosen boundary conditions enforce that all spines along $[0, L]$ get supplied with at least $\phi\eta_p$ proteins, $\phi \in [0, 1)$. We calculated the numerical steady-state solution for (eqs. 40, 41) as described above.

Temporal evolution. During photoactivation ($t > 0$), fluorescent proteins enter the dendritic shaft and, from there, the spines. Note that for all $t \geq 0$, the total amount of proteins (non-photoactivated plus photoactivated) is constant in the dendrite and spines. Therefore, the total protein number always equals its initial value:

$$\begin{aligned} p(t,x) + \tilde{p}(t,x) &= p(0,x), \\ p_{\text{spine}}(t,x) + \tilde{p}_{\text{spine}}(t,x) &= p_{\text{spine}}(0,x). \end{aligned} \tag{42}$$

Thus, the spread of photoactivated proteins determines the distribution of non-photoactivated proteins. The dynamics of photoactivated proteins are given through

$$\partial_t \tilde{p} = D_p \frac{\partial^2}{\partial x^2} \tilde{p} - \lambda_p \tilde{p} - u_p \tilde{p} \underbrace{\left(1 - \frac{p_{\text{spine}} + \tilde{p}_{\text{spine}}}{\rho \eta_p}\right)}_{const.} + \nu_p \tilde{p}_{\text{spine}}, \tag{43a}$$

$$\partial_t \tilde{p}_{\text{spine}} = u_p \tilde{p} \underbrace{\left(1 - \frac{p_{\text{spine}} + \tilde{p}_{\text{spine}}}{\rho \eta_p}\right)}_{const.} - \nu_p \tilde{p}_{\text{spine}} - \lambda_p \tilde{p}_{\text{spine}}. \tag{43b}$$

At the soma, ref.[44] continuously photoactivated somatic *Shank3* with steady laser pulses every 6-10 minutes. Following the authors' suggestions, we assumed all proteins at $x = 0$ get labelled with each somatic labelling pulse: $p(t, 0) = 0$ for $t > 0$. Consequently, we applied the following boundary conditions:

$$\tilde{p}(t,0) = \begin{cases} 0 & t = 0 \\ p(0) & t > 0 \end{cases}, \tag{44a}$$

$$\frac{\partial}{\partial x} \tilde{p}(t, L) = 0 \quad \forall t \geq 0. \tag{44b}$$

In contrast to the initial baseline, the temporal dynamics depend on the integration rate u_p and exit rate ν_p themselves, as well as the assumed supply rate $\phi \in [0, 1)$ and the spine/PSD capacity η_p.

Discretisation. We solved the system (eqs. 43, 44) numerically on $[0, L] \times [0, t_{max}]$ using a finite difference method. We applied an explicit forward-in-time-central-in-space and an implicit backward-in-time-central-in-space scheme on a spatial and temporal grid. Both solutions coincided.

Capturing active protein transport in our model

Here, we describe how our model considered the motor-mediated microtubular transport of proteins along the longitudinal axis of dendrites. Active protein transport in dendrites has been reported for selected protein species such as membrane-bound receptors [43,69–72] along with the caveat that the linear protein motion which is commonly taken as an indication of active protein transport may not always indicate motor-driven motion[73]. The currently available information on motor-mediated active transport of proteins suggests that dendritic protein transport parameters are similar to those of mRNA transport[43,70,72]. Therefore, we applied our mRNA transport model as

described above to protein transport and used the same parameter set for both. The same applies to the associated transport costs.

We examined the energetic efficiency of dendritic protein transport throughout our parameter space while recalculating the mRNA and protein distributions and the related energy costs for all our sampled synthetic protein species (N=3^7=2187). Each synthetic protein species, represented by an individual set of mRNA and protein parameters, had in total four trafficking options: 1) somatic mRNA with no active transport at all, 2) somatic mRNA with protein transport but no mRNA transport, 3) dendritic mRNA with mRNA transport but no protein transport, and 4) dendritic mRNA with mRNA and protein transport. We then compared the cost of the four possible transport schemes for each synthetic protein species. Notably, we found that in our energy optimisation framework, protein transport was always an 'add-on' to mRNA transport because it was only energy-optimal for protein species already favouring dendritic mRNA localisation (Supplementary Figure 3). Second, unlike mRNA transport, we found that protein transport can be an energy-optimal solution only for long dendrites (Supplementary Figure 3). In dendrites equal to or less than 750μm, as in f.e. hippocampal cell cultures (Supplementary Figure 11), protein transport was energy efficient only for very few protein species (Supplementary Figure 3). This result persisted even when five instead of one protein were trafficked together in one vesicle. Finally, we observed that the active transport of proteins (but not of mRNAs) shifted the location of energy consumption into dendrites (Supplementary Figure 4), which is not consistent with the experimentally reported mitochondrial distribution (Figure 1F). We therefore decided to focus on the energetic efficiency of dendritic vs. somatic mRNA localisation in the absence of protein transport. Here, it is essential to note that while our model suggests that active protein transport is not an energy-efficient strategy for maintaining stable copy numbers across synapses (proteostasis), it does not exclude the possibility that active protein transport serves temporally or spatially dynamic synaptic plasticity needs or mediates spine-dendrite coupling [157].

Dataset selection for validation of model predictions

In selecting the databases and matching proteins and mRNAs across databases, we have indeed evaluated multiple database pairings whereby some database pairings had much larger overlaps than others. In selecting databases for Figure 3 we gave preference to the databases which met two criteria: 1) had a large number of entries and 2) had the largest overlap with other databases, e.g., a database reporting half-lives vs a database reporting soma/dendrite localisation ratios needed to have a large overlap in molecular species. A summary of the datasets we considered can be found in Supplementary Table 7. We have cross-matched genes from datasets reporting one of the parameters of interest with datasets that reported other parameters of interest for the same gene (e.g., mRNA neurite-to-soma enrichment scores vs half-lives). To maintain consistency, we have used the same dataset for analysis whenever possible and gave preference to dataset combinations with the highest overlap. Shown in dark gray in Supplementary Table 7 are the cross-matched datasets we selected for Figure 3 based on the database overlap and total sample size. Similarly, dataset pairs highlighted in light gray were used to further confirm our analysis of Figure 3, shown in Supplementary Figure 17. Moreover, a summary of all datasets used in this study can be found in Supplementary Tables 1, 2, 6 along with Supplementary Table 7. We need to emphasize that the number of cross-matching entries does not necessarily mean the final number used for further analysis, since after applying in-depth filters (e.g. removing mRNA half-lives longer than 24h, not recorded entries, and etc), the numbers even drop more and eventually the ones left are categorized into somata- or neurite-enriched category.

Supplementary References

[1] Price, J. C., Guan, S., Burlingame, A., Prusiner, S. B. & Ghaemmaghami, S. Analysis of proteome dynamics in the mouse brain. *Proceedings of the National Academy of Sciences* **107**, 14508–14513 (2010).

[2] Fornasiero, E. F. *et al.* Precisely measured protein lifetimes in the mouse brain reveal differences across tissues and subcellular fractions. *Nature Communications* **9**, 4230 (2018).

[3] Dörrbaum, A. R., Kochen, L., Langer, J. D. & Schuman, E. M. Local and global influences on protein turnover in neurons and glia. *eLife* **7**, e34202 (2018).

[4] Mathieson, T. *et al.* Systematic analysis of protein turnover in primary cells. *Nature Communications* **9**, 689 (2018).

[5] Helm, M. S. *et al.* A large-scale nanoscopy and biochemistry analysis of postsynaptic dendritic spines. *Nature Neuroscience* **24**, 1151–1162 (2021).

[6] Schwanhäusser, B. *et al.* Global quantification of mammalian gene expression control. *Nature* **473**, 337–342 (2011).

[7] Yang, E. *et al.* Decay Rates of Human mRNAs: Correlation With Functional Characteristics and Sequence Attributes. *Genome Research* **13**, 1863–1872 (2003).

[8] Sharova, L. V. *et al.* Database for mRNA Half-Life of 19 977 Genes Obtained by DNA Microarray Analysis of Pluripotent and Differentiating Mouse Embryonic Stem Cells. *DNA Research* **16**, 45–58 (2009).

[9] Tushev, G. *et al.* Alternative 3' UTRs Modify the Localization, Regulatory Potential, Stability, and Plasticity of mRNAs in Neuronal Compartments. *Neuron* **98**, 495–511 (2018).

[10] Buxbaum, A. R., Haimovich, G. & Singer, R. H. In the right place at the right time: visualizing and understanding mRNA localization. *Nature Reviews Molecular Cell Biology* **16**, 95–109 (2014).

[11] Fonkeu, Y. *et al.* How mRNA Localization and Protein Synthesis Sites Influence Dendritic Protein Distribution and Dynamics. *Neuron* **103**, 1109–1122.e7 (2019).

[12] Huang, Y.-S., Carson, J. H., Barbarese, E. & Richter, J. D. Facilitation of dendritic mRNA transport by CPEB. *Genes & Development* **17**, 638–653 (2003).

[13] Park, H. Y. *et al.* Visualization of Dynamics of Single Endogenous mRNA Labeled in Live Mouse. *Science* **343**, 422–424 (2014).

[14] Wang, C., Han, B., Zhou, R. & Zhuang, X. Real-Time Imaging of Translation on Single mRNA Transcripts in Live Cells. *Cell* **165**, 990–1001 (2016).

[15] Dynes, J. L. & Steward, O. Dynamics of bidirectional transport ofArc mRNA in neuronal dendrites. *The Journal of Comparative Neurology* **500**, 433–447 (2006).

[16] Zappulo, A. *et al.* RNA localization is a key determinant of neurite-enriched proteome. *Nature Communications* **8**, 583 (2017).

[17] Cuntz, H., Forstner, F., Borst, A. & Häusser, M. One Rule to Grow Them All: A General Theory of Neuronal Branching and Its Practical Application. *PLoS Computational Biology* **6**, e1000877 (2010).

[18] Kumari, P., Srinivasan, B. & Banerjee, S. Modulation of hippocampal synapse maturation by activity-regulated E3 ligase via non-canonical pathway. *Neuroscience* **364**, 226–241 (2017).

[19] Andreae, L. C. & Burrone, J. Spontaneous Neurotransmitter Release Shapes Dendritic Arbors via Long-Range Activation of NMDA Receptors. *Cell Reports* **10**, 873–882 (2015).

[20] Shirinpour, S. *et al.* Multi-scale modeling toolbox for single neuron and subcellular activity under Transcranial Magnetic Stimulation. *Brain Stimulation* **14**, 1470–1482 (2021).

[21] Chapleau, C. A. *et al.* Dendritic spine pathologies in hippocampal pyramidal neurons from Rett syndrome brain and after expression of Rett-associated MECP2 mutations. *Neurobiology of Disease* **35**, 219–233 (2009).

[22] Harb, A. *et al.* Auxiliary subunits regulate the dendritic turnover of ampa receptors in mouse hippocampal neurons. *Frontiers in molecular neuroscience* **14**, 728498 (2021).

[23] Mikasova, L. *et al.* Disrupted surface cross-talk between NMDA and Ephrin-B2 receptors in anti-NMDA encephalitis. *Brain* **135**, 1606–1621 (2012).

[24] Glock, C. *et al.* The translatome of neuronal cell bodies, dendrites, and axons. *Proceedings of the National Academy of Sciences* **118**, e2113929118 (2021).

[25] Loedige, I. *et al.* mRNA stability and m6A are major determinants of subcellular mRNA localization in neurons. *Molecular Cell* **83**, 2709–2725 (2023).

[26] Farris, S. *et al.* Hippocampal subregions express distinct dendritic transcriptomes that reveal differences in mitochondrial function in ca2. *Cell reports* **29**, 522–539 (2019).

[27] Cajigas, I. J. *et al.* The Local Transcriptome in the Synaptic Neuropil Revealed by Deep Sequencing and High-Resolution Imaging. *Neuron* **74**, 453–466 (2012).

[28] Heo, S. *et al.* Identification of long-lived synaptic proteins by proteomic analysis of synaptosome protein turnover. *Proceedings of the National Academy of Sciences* **115** (2018).

[29] Ensemble BioMart. https://www.ensembl.org/index.html.

[30] Peng, H. B., Zhao, D.-Y., Xie, M.-Z., Shen, Z. & Jacobson, K. The role of lateral migration in the formation of acetylcholine receptor clusters induced by basic polypeptide-coated latex beads. *Developmental biology* **131**, 197–206 (1989).

[31] Hannezo, E., Dong, B., Recho, P., Joanny, J.-F. & Hayashi, S. Cortical instability drives periodic supracellular actin pattern formation in epithelial tubes. *Proceedings of the National Academy of Sciences* **112**, 8620–8625 (2015).

[32] Pelassa, I., Zhao, C., Pasche, M., Odermatt, B. & Lagnado, L. Synaptic vesicles are âœprimedâ for fast clathrin-mediated endocytosis at the ribbon synapse. *Frontiers in Molecular Neuroscience* **7** (2014).

[33] Muir, J. & Kittler, J. T. Plasticity of gabaa receptor diffusion dynamics at the axon initial segment. *Frontiers in cellular neuroscience* **8**, 151 (2014).

[34] Hausrat, T. J. *et al.* Radixin regulates synaptic gabaa receptor density and is essential for reversal learning and short-term memory. *Nature communications* **6**, 6872 (2015).

[35] Renner, M., Schweizer, C., Bannai, H., Triller, A. & Lévi, S. Diffusion barriers constrain receptors at synapses. *PloS one* **7**, e43032 (2012).

[36] Bannai, H. *et al.* Activity-dependent tuning of inhibitory neurotransmission based on gabaar diffusion dynamics. *Neuron* **62**, 670–682 (2009).

[37] Ewers, H. *et al.* A septin-dependent diffusion barrier at dendritic spine necks. *PloS one* **9**, e113916 (2014).

[38] Papouin, T. *et al.* Synaptic and extrasynaptic nmda receptors are gated by different endogenous coagonists. *Cell* **150**, 633–646 (2012).

[39] Groc, L. *et al.* NMDA receptor surface mobility depends on NR2A-2B subunits. *Proceedings of the National Academy of Sciences* **103**, 18769–18774 (2006).

[40] Sergé, A., Fourgeaud, L., Hémar, A. & Choquet, D. Receptor activation and homer differentially control the lateral mobility of metabotropic glutamate receptor 5 in the neuronal membrane. *Journal of Neuroscience* **22**, 3910–3920 (2002).

[41] Gil, O. D. *et al.* Ankyrin binding mediates l1cam interactions with static components of the cytoskeleton and inhibits retrograde movement of l1cam on the cell surface. *The Journal of cell biology* **162**, 719–730 (2003).

[42] Boiko, T. *et al.* Ankyrin-dependent and-independent mechanisms orchestrate axonal compartmentalization of l1 family members neurofascin and l1/neuron–glia cell adhesion molecule. *Journal of Neuroscience* **27**, 590–603 (2007).

[43] Neupert, C. *et al.* Regulated Dynamic Trafficking of Neurexins Inside and Outside of Synaptic Terminals. *The Journal of Neuroscience* **35**, 13629–13647 (2015).

[44] Tsuriel, S. *et al.* Local Sharing as a Predominant Determinant of Synaptic Matrix Molecular Dynamics. *PLoS Biology* **4**, e271 (2006).

[45] Bateman, A. *et al.* UniProt: the Universal Protein Knowledgebase in 2023. *Nucleic Acids Research* **51**, D523–D531 (2023).

[46] Perez, J. D. *et al.* Subcellular sequencing of single neurons reveals the dendritic transcriptome of GABAergic interneurons. *eLife* **10**, e63092 (2021).

[47] Zeisel, A. *et al.* Cell types in the mouse cortex and hippocampus revealed by single-cell RNA-seq. *Science* **347**, 1138–1142 (2015).

[48] Fusco, D. *et al.* Single mRNA Molecules Demonstrate Probabilistic Movement in Living Mammalian Cells. *Current Biology* **13**, 161–167 (2003).

[49] Knowles, R. B. *et al.* Translocation of RNA Granules in Living Neurons. *The Journal of Neuroscience* **16**, 7812–7820 (1996).

[50] Rook, M. S., Lu, M. & Kosik, K. S. CaMKIIα 3 Untranslated Region-Directed mRNA Translocation in Living Neurons: Visualization by GFP Linkage. *The Journal of Neuroscience* **20**, 6385–6393 (2000).

[51] Elvira, G. *et al.* Characterization of an RNA Granule from Developing Brain. *Molecular & Cellular Proteomics* **5**, 635–651 (2006).

[52] Tubing, F. *et al.* Dendritically Localized Transcripts Are Sorted into Distinct Ribonucleoprotein Particles That Display Fast Directional Motility along Dendrites of Hippocampal Neurons. *Journal of Neuroscience* **30**, 4160–4170 (2010).

[53] Tiruchinapalli, D. M. *et al.* Activity-Dependent Trafficking and Dynamic Localization of Zipcode Binding Protein 1 and β-Actin mRNA in Dendrites and Spines of Hippocampal Neurons. *The Journal of Neuroscience* **23**, 3251–3261 (2003).

[54] McKenney, R. J., Huynh, W., Tanenbaum, M. E., Bhabha, G. & Vale, R. D. Activation of cytoplasmic dynein motility by dynactin-cargo adapter complexes. *Science* **345**, 337–341 (2014).

[55] Mitsumori, K., Takei, Y. & Hirokawa, N. Components of RNA granules affect their localization and dynamics in neuronal dendrites. *Molecular Biology of the Cell* **28**, 1412–1417 (2017).

[56] Donlin-Asp, P. G., Polisseni, C., Klimek, R., Heckel, A. & Schuman, E. M. Differential regulation of local mRNA dynamics and translation following long-term potentiation and depression. *Proceedings of the National Academy of Sciences* **118**, e2017578118 (2021).

[57] Köhrmann, M. *et al.* Microtubule-dependent Recruitment of Staufen-Green Fluorescent Protein into Large RNA-containing Granules and Subsequent Dendritic Transport in Living Hippocampal Neurons. *Molecular Biology of the Cell* **10**, 2945–2953 (1999).

[58] Yoon, Y. J. *et al.* Glutamate-induced RNA localization and translation in neurons. *Proceedings of the National Academy of Sciences* **113**, E6877–E6886 (2016).

[59] Kon, T., Nishiura, M., Ohkura, R., Toyoshima, Y. Y. & Sutoh, K. Distinct Functions of Nucleotide-Binding/Hydrolysis Sites in the Four AAA Modules of Cytoplasmic Dynein. *Biochemistry* **43**, 11266–11274 (2004).

[60] Kural, C. *et al.* Kinesin and Dynein Move a Peroxisome in Vivo: A Tug-of-War or Coordinated Movement? *Science* **308**, 1469–1472 (2005).

[61] Lakadamyali, M., Rust, M. J., Babcock, H. P. & Zhuang, X. Visualizing infection of individual influenza viruses. *Proceedings of the National Academy of Sciences* **100**, 9280–9285 (2003).

[62] Ma, S. & Chisholm, R. L. Cytoplasmic dynein-associated structures move bidirectionally in vivo. *Journal of Cell Science* **115**, 1453–1460 (2002).

[63] Ori-McKenney, K. M., Xu, J., Gross, S. P. & Vallee, R. B. A cytoplasmic dynein tail mutation impairs motor processivity. *Nature Cell Biology* **12**, 1228–1234 (2010).

[64] Monnier, N. *et al.* Inferring transient particle transport dynamics in live cells. *Nature Methods* **12**, 838–840 (2015).

[65] Bauer, K. E. *et al.* Live cell imaging reveals 3-UTR dependent mRNA sorting to synapses. *Nature Communications* **10** (2019).

[66] Das, S., Moon, H. C., Singer, R. H. & Park, H. Y. A transgenic mouse for imaging activity-dependent dynamics of endogenous Arc mRNA in live neurons. *Science Advances* **4**, eaar3448 (2018).

[67] Zimyanin, V. L. *et al.* In Vivo Imaging of oskar mRNA Transport Reveals the Mechanism of Posterior Localization. *Cell* **134**, 843–853 (2008).

[68] Amrute-Nayak, M. & Bullock, S. L. Single-molecule assays reveal that RNA localization signals regulate dyneindynactin copy number on individual transcript cargoes. *Nature Cell Biology* **14**, 416–423 (2012).

[69] Guillaud, L., Setou, M. & Hirokawa, N. KIF17 Dynamics and Regulation of NR2B Trafficking in Hippocampal Neurons. *The Journal of Neuroscience* **23**, 131–140 (2003).

[70] Hangen, E., Cordelières, F. P., Petersen, J. D., Choquet, D. & Coussen, F. Neuronal Activity and Intracellular Calcium Levels Regulate Intracellular Transport of Newly Synthesized AMPAR. *Cell Reports* **24**, 1001–1012.e3 (2018).

[71] Setou, M., Nakagawa, T., Seog, D.-H. & Hirokawa, N. Kinesin Superfamily Motor Protein KIF17 and mLin-10 in NMDA Receptor-Containing Vesicle Transport. *Science* **288**, 1796–1802 (2000).

[72] Song, A.-h. *et al.* A Selective Filter for Cytoplasmic Transport at the Axon Initial Segment. *Cell* **136**, 1148–1160 (2009).

[73] Jaqaman, K. *et al.* Cytoskeletal Control of CD36 Diffusion Promotes Its Receptor and Signaling Function. *Cell* **146**, 593–606 (2011).

[74] Hawkin, J. D. A survey on intron and exon lengths. *Nucleic Acids Research* **16**, 9893–9908 (1988).

[75] Lynch, M. & Marinov, G. K. The bioenergetic costs of a gene. *Proceedings of the National Academy of Sciences* **112**, 15690–15695 (2015).

[76] Roy, M., Kim, N., Xing, Y. & Lee, C. The effect of intron length on exon creation ratios during the evolution of mammalian genomes. *RNA* **14**, 2261–2273 (2008).

[77] Batish, M., van den Bogaard, P., Kramer, F. R. & Tyagi, S. Neuronal mRNAs travel singly into dendrites. *Proceedings of the National Academy of Sciences* **109**, 4645–4650 (2012).

[78] Farris, S., Lewandowski, G., Cox, C. D. & Steward, O. Selective Localization of Arc mRNA in Dendrites Involves Activity- and Translation-Dependent mRNA Degradation. *Journal of Neuroscience* **34**, 4481–4493 (2014).

[79] Krichevsky, A. M. & Kosik, K. S. Neuronal RNA Granules. *Neuron* **32**, 683–696 (2001).

[80] Barbarese, E. *et al.* Protein translation components are colocalized in granules in oligodendrocytes. *Journal of Cell Science* **108**, 2781–2790 (1995).

[81] Obashi, K., Matsuda, A., Inoue, Y. & Okabe, S. Precise Temporal Regulation of Molecular Diffusion within Dendritic Spines by Actin Polymers during Structural Plasticity. *Cell Reports* **27**, 1503–1515.e8 (2019).

[82] Yan, X., Hoek, T. A., Vale, R. D. & Tanenbaum, M. E. Dynamics of Translation of Single mRNA Molecules In˜Vivo. *Cell* **165**, 976–989 (2016).

[83] Kanai, Y., Dohmae, N. & Hirokawa, N. Kinesin Transports RNA. *Neuron* **43**, 513–525 (2004).

[84] Kapitein, L. C. *et al.* Mixed Microtubules Steer Dynein-Driven Cargo Transport into Dendrites. *Current Biology* **20**, 290–299 (2010).

[85] Bressloff, P. C. & Newby, J. M. Stochastic models of intracellular transport. *Reviews of Modern Physics* **85**, 135–196 (2013).

[86] Kunwar, A. *et al.* Mechanical stochastic tug-of-war models cannot explain bidirectional lipid-droplet transport. *Proceedings of the National Academy of Sciences* **108**, 18960–18965 (2011).

[87] Muller, M. J. I., Klumpp, S. & Lipowsky, R. Tug-of-war as a cooperative mechanism for bidirectional cargo transport by molecular motors. *Proceedings of the National Academy of Sciences* **105**, 4609–4614 (2008).

[88] Williams, A. H., ODonnell, C., Sejnowski, T. J. & OLeary, T. Dendritic trafficking faces physiologically critical speed-precision tradeoffs. *eLife* **5** (2016).

[89] Burton, P. R. Dendrites of mitral cell neurons contain microtubules of opposite polarity. *Brain Research* **473**, 107–115 (1988).

[90] Baas, P. W., Deitch, J. S., Black, M. M. & Banker, G. A. Polarity orientation of microtubules in hippocampal neurons: uniformity in the axon and nonuniformity in the dendrite. *Proceedings of the National Academy of Sciences* **85**, 8335–8339 (1988).

[91] Yau, K. W. *et al.* Dendrites In Vitro and In Vivo Contain Microtubules of Opposite Polarity and Axon Formation Correlates with Uniform Plus-End-Out Microtubule Orientation. *Journal of Neuroscience* **36**, 1071–1085 (2016).

[92] Glock, C., Heumüller, M. & Schuman, E. M. mRNA transport & local translation in neurons. *Current Opinion in Neurobiology* **45**, 169–177 (2017).

[93] Kiebler, M. A. & Bassell, G. J. Neuronal RNA Granules: Movers and Makers. *Neuron* **51**, 685–690 (2006).

[94] Zeitelhofer, M., Macchi, P. & Dahm, R. Perplexing bodies: The putative roles of P-bodies in neurons. *RNA Biology* **5**, 244–248 (2008).

[95] Mikl, M., Vendra, G. & Kiebler, M. A. Independent localization of MAP2 , CaMKIIα and β-actin RNAs in low copy numbers. *EMBO reports* **12**, 1077–1084 (2011).

[96] Bertrand, E. *et al.* Localization of ASH1 mRNA Particles in Living Yeast. *Molecular Cell* **2**, 437–445 (1998).

[97] Cha, B.-J., Koppetsch, B. S. & Theurkauf, W. E. In Vivo Analysis of Drosophila bicoid mRNA Localization Reveals a Novel Microtubule-Dependent Axis Specification Pathway. *Cell* **106**, 35–46 (2001).

[98] Kanai, Y., Dohmae, N. & Hirokawa, N. Kinesin transports rna: Isolation and characterization of an rna-transporting granule. *Neuron* **43**, 513–525 (2004).

[99] Bauer, K. E., de Queiroz, B. R., Kiebler, M. A. & Besse, F. Rna granules in neuronal plasticity and disease. *Trends in Neurosciences* **46**, 525–538 (2023).

[100] Ripin, N. & Parker, R. Formation, function, and pathology of rnp granules. *Cell* **186**, 4737–4756 (2023).

[101] Sartori, F. *et al.* Statistical Laws of Protein Motion in Neuronal Dendritic Trees. *Cell Reports* **33**, 108391 (2020).

[102] Ehlers, M. D. Activity level controls postsynaptic composition and signaling via the ubiquitin-proteasome system. *Nature Neuroscience* **6**, 231–242 (2003).

[103] Pak, D. T. S. & Sheng, M. Targeted Protein Degradation and Synapse Remodeling by an Inducible Protein Kinase. *Science* **302**, 1368–1373 (2003).

[104] Triesch, J., Vo, A. D. & Hafner, A.-S. Competition for synaptic building blocks shapes synaptic plasticity. *eLife* **7**, e37836 (2018).

[105] Sutton, M. A. & Schuman, E. M. Dendritic Protein Synthesis, Synaptic Plasticity, and Memory. *Cell* **127**, 49–58 (2006).

[106] Floor, S. N. & Doudna, J. A. Tunable protein synthesis by transcript isoforms in human cells. *eLife* **5**, e10921 (2016).

[107] Wu, B., Eliscovich, C., Yoon, Y. J. & Singer, R. H. Translation dynamics of single mRNAs in live cells and neurons. *Science* **352**, 1430–1435 (2016).

[108] Doyle, M. & Kiebler, M. A. Mechanisms of dendritic mRNA transport and its role in synaptic tagging. *The EMBO Journal* **30**, 3540–3552 (2011).

[109] Erickson, S. L. & Lykke-Andersen, J. Cytoplasmic mRNP granules at a glance. *Journal of Cell Science* **124**, 293–297 (2011).

[110] Hüttelmaier, S. *et al.* Spatial regulation of β-actin translation by Src-dependent phosphorylation of ZBP1. *Nature* **438**, 512–515 (2005).

[111] Sonneveld, S., Verhagen, B. M. P. & Tanenbaum, M. E. Heterogeneity in mRNA Translation. *Trends in Cell Biology* **30**, 606–618 (2020).

[112] Morisaki, T. *et al.* Real-time quantification of single RNA translation dynamics in living cells. *Science* **352**, 1425–1429 (2016).

[113] Riba, A. *et al.* Protein synthesis rates and ribosome occupancies reveal determinants of translation elongation rates. *Proceedings of the National Academy of Sciences* **116**, 15023–15032 (2019).

[114] Jacobs, B. Regional Dendritic and Spine Variation in Human Cerebral Cortex: a Quantitative Golgi Study. *Cerebral Cortex* **11**, 558–571 (2001).

[115] Elston, G. N. A Study of Pyramidal Cell Structure in the Cingulate Cortex of the Macaque Monkey with Comparative Notes on Inferotemporal and Primary Visual Cortex. *Cerebral Cortex* **15**, 64–73 (2004).

[116] Elston, G. N. The Pyramidal Cell of the Sensorimotor Cortex of the Macaque Monkey: Phenotypic Variation. *Cerebral Cortex* **12**, 1071–1078 (2002).

[117] Elston, G. N., Benavides-Piccione, R. & DeFelipe, J. The Pyramidal Cell in Cognition: A Comparative Study in Human and Monkey. *The Journal of Neuroscience* **21**, RC163–RC163 (2001).

[118] Ballesteros-Yáñez, I., Benavides-Piccione, R., Elston, G. N., Yuste, R. & DeFelipe, J. Density and morphology of dendritic spines in mouse neocortex. *Neuroscience* **138**, 403–409 (2006).

[119] Brandt, N., Löffler, T., Fester, L. & Rune, G. M. Sex-specific features of spine densities in the hippocampus. *Scientific Reports* **10**, 11405 (2020).

[120] An, J. J. *et al.* Distinct Role of Long 3 UTR BDNF mRNA in Spine Morphology and Synaptic Plasticity in Hippocampal Neurons. *Cell* **134**, 175–187 (2008).

[121] Kretz, O. Hippocampal Synapses Depend on Hippocampal Estrogen Synthesis. *Journal of Neuroscience* **24**, 5913–5921 (2004).

[122] Bannister, N. J. & Larkman, A. U. Dendritic morphology of CA1 pyramidal neurones from the rat hippocampus: II. Spine distributions. *The Journal of Comparative Neurology* **360**, 161–171 (1995).

[123] Prange-Kiel, J. *et al.* Gonadotropin-releasing hormone regulates spine density via its regulatory role in hippocampal estrogen synthesis. *Journal of Cell Biology* **180**, 417–426 (2008).

[124] Ashby, M. C., Maier, S. R., Nishimune, A. & Henley, J. M. Lateral Diffusion Drives Constitutive Exchange of AMPA Receptors at Dendritic Spines and Is Regulated by Spine Morphology. *Journal of Neuroscience* **26**, 7046–7055 (2006).

[125] Sharma, K., Fong, D. K. & Craig, A. M. Postsynaptic protein mobility in dendritic spines: Long-term regulation by synaptic NMDA receptor activation. *Molecular and Cellular Neuroscience* **31**, 702–712 (2006).

[126] Svoboda, K., Tank, D. W. & Denk, W. Direct Measurement of Coupling Between Dendritic Spines and Shafts. *Science* **272**, 716–719 (1996).

[127] Simon, C. M., Hepburn, I., Chen, W. & Schutter, E. D. The role of dendritic spine morphology in the compartmentalization and delivery of surface receptors. *Journal of Computational Neuroscience* **36**, 483–497 (2013).

[128] Trimble, W. S. & Grinstein, S. Barriers to the free diffusion of proteins and lipids in the plasma membrane. *Journal of Cell Biology* **208**, 259–271 (2015).

[129] Byrne, M. J., Waxham, M. N. & Kubota, Y. The impacts of geometry and binding on CaMKII diffusion and retention in dendritic spines. *Journal of Computational Neuroscience* **31**, 1–12 (2010).

[130] Holcman, D. & Schuss, Z. Diffusion laws in dendritic spines. *The Journal of Mathematical Neuroscience* **1**, 10 (2011).

[131] Kusters, R., Kapitein, L. C., Hoogenraad, C. C. & Storm, C. Shape-Induced Asymmetric Diffusion in Dendritic Spines Allows Efficient Synaptic AMPA Receptor Trapping. *Biophysical Journal* **105**, 2743–2750 (2013).

[132] Khan, S., Reese, T. S., Rajpoot, N. & Shabbir, A. Spatiotemporal maps of CaMKII in dendritic spines. *Journal of Computational Neuroscience* **33**, 123–139 (2012).

[133] Honkura, N., Matsuzaki, M., Noguchi, J., Ellis-Davies, G. C. R. & Kasai, H. The Subspine Organization of Actin Fibers Regulates the Structure and Plasticity of Dendritic Spines. *Neuron* **57**, 719–729 (2008).

[134] Lee, S.-J. R., Escobedo-Lozoya, Y., Szatmari, E. M. & Yasuda, R. Activation of CaMKII in single dendritic spines during long-term potentiation. *Nature* **458**, 299–304 (2009).

[135] Dupuis, J. P. *et al.* Surface dynamics of GluN2B-NMDA receptors controls plasticity of maturing glutamate synapses. *The EMBO Journal* **33**, 842–861 (2014).

[136] Attwell, D. & Laughlin, S. B. An Energy Budget for Signaling in the Grey Matter of the Brain. *Journal of Cerebral Blood Flow & Metabolism* **21**, 1133–1145 (2001).

[137] Hu, X.-P., Dourado, H., Schubert, P. & Lercher, M. J. The protein translation machinery is expressed for maximal efficiency in Escherichia coli. *Nature Communications* **11**, 5260 (2020).

[138] Moldave, K. Eukaryotic Protein Synthesis. *Annual Review of Biochemistry* **54**, 1109–1149 (1985).

[139] Luby-Phelps, K. Cytoarchitecture and Physical Properties of Cytoplasm: Volume, Viscosity, Diffusion, Intracellular Surface Area. In *International Review of Cytology*, 189–221 (Elsevier, 1999).

[140] Kural, C. *et al.* Tracking melanosomes inside a cell to study molecular motors and their interaction. *Proceedings of the National Academy of Sciences* **104**, 5378–5382 (2007).

[141] Chang, L. & Goldman, R. D. Intermediate filaments mediate cytoskeletal crosstalk. *Nature Reviews Molecular Cell Biology* **5**, 601–613 (2004).

[142] Lau, A. W. C., Hoffman, B. D., Davies, A., Crocker, J. C. & Lubensky, T. C. Microrheology, Stress Fluctuations, and Active Behavior of Living Cells. *Physical Review Letters* **91**, 198101 (2003).

[143] Brangwynne, C. P., Koenderink, G. H., MacKintosh, F. C. & Weitz, D. A. Cytoplasmic diffusion: molecular motors mix it up. *Journal of Cell Biology* **183**, 583–587 (2008).

[144] Bursac, P. *et al.* Cytoskeletal remodelling and slow dynamics in the living cell. *Nature Materials* **4**, 557–561 (2005).

[145] Mallik, R., Carter, B. C., Lex, S. A., King, S. J. & Gross, S. P. Cytoplasmic dynein functions as a gear in response to load. *Nature* **427**, 649–652 (2004).

[146] Svoboda, K., Schmidt, C. F., Schnapp, B. J. & Block, S. M. Direct observation of kinesin stepping by optical trapping interferometry. *Nature* **365**, 721–727 (1993).

[147] Hua, W., Young, E. C., Fleming, M. L. & Gelles, J. Coupling of kinesin steps to ATP hydrolysis. *Nature* **388**, 390–393 (1997).

[148] Schnitzer, M. J. & Block, S. M. Kinesin hydrolyses one ATP per 8-nm step. *Nature* **388**, 386–390 (1997).

[149] Vale, R. D. & Milligan, R. A. The Way Things Move: Looking Under the Hood of Molecular Motor Proteins. *Science* **288**, 88–95 (2000).

[150] Yildiz, A., Tomishige, M., Vale, R. D. & Selvin, P. R. Kinesin Walks Hand-Over-Hand. *Science* **303**, 676–678 (2004).

[151] Kulic, I. M. *et al.* The role of microtubule movement in bidirectional organelle transport. *Proceedings of the National Academy of Sciences* **105**, 10011–10016 (2008).

[152] Epstein, I. *et al.* Alternative polyadenylation and differential expression of Shank mRNAs in the synaptic neuropil. *Philosophical Transactions of the Royal Society B: Biological Sciences* **369**, 20130137 (2014).

[153] Lowenthal, M. S., Markey, S. P. & Dosemeci, A. Quantitative Mass Spectrometry Measurements Reveal Stoichiometry of Principal Postsynaptic Density Proteins. *Journal of Proteome Research* **14**, 2528–2538 (2015).

[154] Chen, X. *et al.* Mass of the postsynaptic density and enumeration of three key molecules. *Proceedings of the National Academy of Sciences* **102**, 11551–11556 (2005).

[155] Cheng, D. *et al.* Relative and Absolute Quantification of Postsynaptic Density Proteome Isolated from Rat Forebrain and Cerebellum. *Molecular and Cellular Proteomics* **5**, 1158–1170 (2006).

[156] Peng, J. *et al.* Semiquantitative Proteomic Analysis of Rat Forebrain Postsynaptic Density Fractions by Mass Spectrometry. *Journal of Biological Chemistry* **279**, 21003–21011 (2004).

[157] McVicker, D., Awe, A., Richters, K. & et al. Transport of a kinesin-cargo pair along microtubules into dendritic spines undergoing synaptic plasticity. *Nature Communications* **7**, 12741 (2016).
